# Emotional and behavioral outcomes among youths with mental disorders during the first Covid lockdown and school closures in England: a large clinical population study using health care record integrated surveys

**DOI:** 10.1007/s00127-023-02517-w

**Published:** 2023-06-23

**Authors:** V. Parlatini, L. Frangou, S. Zhang, S. Epstein, A. Morris, C. Grant, L. Zalewski, A. Jewell, S. Velupillai, E. Simonoff, J. Downs

**Affiliations:** 1 Department of Child and Adolescent Psychiatry, Institute of Psychiatry, Psychology and Neuroscience, King’s College London, 16 De Crespigny Park, London, SE5 8AF UK; 2 Department of Epidemiology and Public Health, University College London, London, UK; 3 Department of Biostatistics and Health Informatics, Institute of Psychiatry, Psychology and Neuroscience, King’s College London, London, UK; 4 National Institute for Health Research (NIHR) Biomedical Research Centre, South London and Maudsley NHS Foundation Trust, London, UK

**Keywords:** Children and young people, Mental disorders, Covid pandemic, Remote education, Survey, Electronic health records

## Abstract

**Purpose:**

Emotional and behavioral problems in children and young people (CYP) have increased over the pandemic. Those with pre-existing mental disorders are more vulnerable but have been understudied. We investigated emotional and behavioral outcomes in this population; differences across diagnostic groups; and social, educational, and clinical determinants.

**Methods:**

We invited 5386 caregivers and CYP (aged 5–17) under child mental health services pre-pandemic to complete an online survey on CYP’s emotional/behavioral symptoms and pandemic-related circumstances, and integrated responses with clinicodemographic information extracted from electronic health records. We compared four parent-rated outcomes (total emotional/behavioral scores and emotional/behavioral changes as compared to before the pandemic) across the three most common diagnostic groups in our population (Attention Deficit Hyperactivity Disorder (ADHD), Autism Spectrum Disorder (ASD) and emotional disorders (EmD)). We then estimated the association of clinicodemographic and pandemic-related characteristics with emotional/behavioral outcomes.

**Results:**

A total of 1741 parents (32.3%) completed the survey. Parents of CYP with ADHD or ASD reported more behavioral difficulties (*t*(591) = 5.618 (0.001); *t*(663) = 6.527 (0.001)); greater emotional deterioration (*t*(591) = 2.592 (0.009); *t*(664) = 4.670 (< 0.001); and greater behavioral deterioration (*t*(594) = 4.529 (< 0.001); *t*(664) = 5.082 (< 0.001)) as compared to the EmD group. Those with ASD and EmD showed more emotional difficulties than ADHD (*t*(891) = − 4.431 (< 0.001); *t*(590) = − 3.254 (0.001)). Across diagnoses, poor parental mental health and challenges with education were most strongly associated with worse outcomes.

**Conclusions:**

Within our clinical population, CYP with ADHD/ASD were the most adversely affected during lockdown. Enhancing clinical service provision that tackles parental stress and supports education may help mitigate the impact of future restrictions.

**Supplementary Information:**

The online version contains supplementary material available at 10.1007/s00127-023-02517-w.

## Introduction

The Covid-19 pandemic has changed many aspects of the lives of children and young people (CYP) globally. Since the outbreak, the government of the United Kingdom (UK) has implemented a series of national lockdowns during which workplaces and community spaces were closed (Supplementary material, Sect.  1.1, and Fig. S1 for timeframe). Schools were also closed to pupils, unless considered vulnerable or children of keyworkers, and education was provided remotely. Although schools gradually reopened from June 2020, disruption has continued due to ongoing restrictions and local outbreaks. Quarantine measures also meant a rapid re-configuration of Child and Adolescent Mental Health Services (CAMHS), which initially suspended most routine in-person appointments to focus on emergency work and/or moved to online appointments.

Concerns have grown regarding the impact of prolonged social distancing measures and school closure on the mental health of previously healthy CYP, as demonstrated by the world-wide increased rates of depression, anxiety, inattention, problematic eating and alcohol and cannabis use [1–4]. This increase has been particularly evident during the first period of lockdown [5]. Disruption of routines, family and peer relationships, education, and support from services were identified as risk factors [6, 7], while a supportive network and adaptive coping strategies as protective factors [4, 6]. There have been fewer studies on CYP with pre-existing mental disorders but the existing ones mostly reported a negative impact, with worsening of pre-existing symptoms and/or emerging of new complaints, which they linked to their vulnerability and pandemic-related reduced support from services [8–12]. However, other studies did not observe worsening but stable or improved symptoms [13–15], which suggests that distinct clinical populations may be differentially affected according to diagnosis, socio-demographic characteristics, and pandemic-related factors. Most prior studies investigating the effects of lockdown included mixed clinical samples [9, 13, 16] or only CYP with a specific diagnosis [8, 12, 17]; and the majority of them focused on neurodevelopmental disorders. Thus, it is unclear whether CYP with distinct diagnoses may have been differentially affected. Further, prior studies including clinical samples reported that age, parental mental health, and financial challenges were associated with worsening of CYP’s symptoms [9, 12, 17]. However, other potential contributing factors, such as ethnicity, housing adequacy, and the type of restrictions, were less investigated. Finally, the relationship between remote education experience and mental health in CYP with pre-existing mental disorders has received limited attention [18, 19], thus the impact of changes in education provision in this vulnerable population is unclear. Addressing these questions is of importance as it may guide policy and clinical practice, and thus help mitigate the effects of this pandemic and inform the response to any future ones. For instance, child mental health services are still unclear about how to organize their limited resources, as there is considerable uncertainty over which diagnostic groups have been most affected; what pre-Covid socio-demographic and pandemic-related contextual factors conferred most risk; and what was the impact of changes in education provision.

This study aims to address these questions and is part of a larger clinical population-based prospective cohort study, which surveyed CYP and families under CAMHS at three time points during the pandemic. This specific report focuses on cross-sectional data based on parent responses to the first survey (June–September 2020) integrated with pre-pandemic socio-demographic and clinical information as routinely recorded on CAMHS electronic health records (EHR).

## Methods

### Sample

The sampling frame included 5386 CYP (aged 5–17) that were active out-patients in community and specialist services within CAMHS at South London and Maudsley NHS Foundation Trust (SLaM), London (UK), on the 1st June 2020. SLaM is one of the biggest providers of mental healthcare in Europe with a catchment area of 1.3 million residents. Additionally, it offers specialist assessments and treatments to CYP from the rest of the UK. The catchment area is ethnically very diverse and includes boroughs with the highest percentage of Afro-Caribbean people in England and Wales (https://www.ethnicity-facts-figures.service.gov.uk). It also has the highest proportions of children in income deprivation in England (https://www.gov.uk/government/statistics/english-indices-of-deprivation-2015). Caregivers and CYP themselves (with parental consent if below 16) were invited to complete the survey (Supplementary Methods, 1.2). We excluded CYP who were admitted at the time, as they represent a minority with particularly severe presentations and specific needs.

### Data source

The Maudsley Child and Young People Health and Experience Research (CYPHER) survey was developed from the widely used questionnaire CoRonavIruS Health Impact Survey (CRISIS) [20] and specifically adapted to the CAMHS clinical population across Europe and Oceania. It included 76 questions for caregivers and 36 for young people and covered mental health symptoms and pandemic-related contextual factors (as summarized below and detailed in the Appendix). The survey was launched at three time points during the pandemic as part of patient monitoring activities under the UK legal framework of Regulation 3(2)/3(3) of the Health Service Control of Patient Information 2002 (COPI). This report focuses on the first wave (responses obtained between 15th June 2020 and 2nd September 2020) and, primarily, on caregiver responses to also include data on children that could not provide responses themselves (e.g., due to young age or intellectual disability—ID). We controlled whether parental mental health potentially introduced a source of bias in a sensitivity analysis (see below). For completeness, young people’s responses to individual survey questions and correlations for matched parent–young person responses are reported in Appendix.

Baseline clinicodemographic data were drawn from CAMHS electronic health records (EHR) and extracted via the Clinical Record Interactive Search (CRIS) tool, a digital platform that de-identifies comprehensive structured and free-text patient data for secondary mental health research [21]. Pre-Covid socio-demographic characteristics included age, sex, ethnicity, and neighborhood deprivation (as detailed in Supplementary Methods, 1.3). Diagnoses were extracted as routinely recorded by clinicians on EHRs based on the International Classification of Diseases 10th edition (ICD‐10) [22] (Supplementary Methods, 1.4). Data extraction was completed on 30th September 2020 for all CYP independently from their participation to the survey, which allowed us to gather socio-clinical information for both responders and non-responders. It was then matched to the available individual survey responses (Fig. S2 for data flow). Extraction and analysis of deidentified data were carried out using the CRIS platform and security model. Overarching ethical approval for secondary data analysis of deidentified data using CRIS was granted by the Oxford Research Ethics Committee C (08/H0606/71 + 5) [23], and this specific project was approved by an oversight committee (N. 20-040, https://projects.slam.nhs.uk/research/cris/cris_projectsdetails?ID=1495).

### Outcomes

We considered four parent-rated outcomes. The survey was developed at hoc to test the effects of the pandemic on CYP’s mental health and did not include a validated scale but 12 questions on child’s symptoms (Appendix). Thus, we used factor analysis to test the underlying structure. This identified two symptom domains, one including emotional symptoms (anhedonia, sadness, general worries, anxiety, fatigue, and loneliness) and one behavioral symptoms (restlessness, inattention, irritability, and aggression). Within the respective domain, Likert scores of individual symptoms were summed to generate a parent-rated total emotional score and total behavioral score (range 6–30 and 5–20, respectively) (Supplementary methods, 1.5). These two composite scores were then used as outcomes. Further, parents were also asked to rate the degree of their child’s symptom change as compared to before the pandemic on a 5-point Likert scale ranging from much better to much worse, which generated two further outcomes (emotional change and behavioral change). For all four outcomes, higher scores reflected increased symptom severity or worsening.

### Pandemic-related contextual factors

Pandemic-related contextual characteristics were derived from parent survey responses. Specifically, we aggregated responses according to their common themes as confirmed with factor analysis (or correlations where the former was not feasible), and calculated corresponding composite scores (Supplementary methods, 1.5). Responses included in each composite score are highlighted in Appendix. Composite scores included housing inadequacy; poor parental mental health; lack of family support; parental concerns regarding finances and housing; challenges with education; and perceived inadequacy of child’s mental health care. The two questions on restrictions, limited outdoor time and difficulty with social distancing, were not significantly correlated and were considered separately.

### Socio-demographic characteristics

Pre-Covid socio-demographic characteristics included age, sex, ethnicity, and neighborhood deprivation (as described in Supplementary methods, 1.3), which were extracted from EHR. We selected these variables as they have been previously investigated in clinical samples [9, 12, 17].

### Statistical analysis

Statistical analyses were conducted using Stata version 15 (StatCorp, USA). We first compared mean differences in outcome measures and pandemic-related contextual characteristics among the three most common diagnostic groups in our population using one-way ANOVA and post hoc t-tests where appropriate.

We then used unadjusted and adjusted multivariable linear regressions to identify pre-Covid socio-demographic and Covid-related contextual factors associated with the four parent-rated outcomes for the whole sample and within diagnostic group. Specifically, pandemic-related contextual characteristics derived from parent survey responses (15th June–2nd September 2020) represented the primary exposure of interest. Pre-Covid socio-demographic characteristics extracted from EHR (30th September 2020) were considered as covariates. In the fully adjusted models for the three groups, we stratified the analyses by the main diagnosis but also included additional disorders (i.e., ADHD, ASD, EmD and ID), if present, as covariates to account for comorbid presentations. We considered ID in addition to the main diagnoses as we hypothesized this may limit the ability to understand the implications of the pandemic but also to adapt to social distancing measures.

Finally, to better understand the impact of changes in education provision, we tested whether the three diagnostic groups differed in remote education enjoyment/engagement or enjoyment according to education modality; and whether education enjoyment/engagement was associated with emotional or behavioral change (Supplementary methods, 1.7).

### Sensitivity analyses

We carried out two sensitivity analyses. The first verified whether the observed association between parent and child mental health was due to a shared-method variance effect; whilst the latter tested whether differences in mental health outcomes among the three main diagnostic groups held independently from contextual and socio-demographic factors (Supplementary methods, 1.6).

## Results

### Sample

We obtained 2503 responses (46.5%). The flow diagram is reported in Fig.S4. As shown in Table 1, responders and non-responders had similar clinicodemographic characteristics. Responders mainly self-identified as White (49.6%) or Black (18.8%) and resided in the least deprived neighborhoods (22.5%). CYP had a mean age of 13.2 (± 3.2) years and were mainly males (55%). The three most common diagnoses were: Attention Deficit Hyperactivity Disorder—ADHD (i.e., hyperkinetic disorders); Autism Spectrum Disorder—ASD; and emotional disorders—EmD (which included depressive and anxiety disorders, Post-Traumatic Stress Disorder—PTSD, and Obsessive–Compulsive Disorder—OCD). Further, 4.3% had ID. (Supplementary methods, 1.4, Table 1). Pre-Covid clinicodemographic characteristics according to diagnostic group are reported in Table S2. In this work, we focused on responses provided by caregivers (*N* = 1741), who mostly self-identified as parents. As shown in the Venn diagram (Fig. S4), 32.7% of CYP were diagnosed with ADHD, 18.3% with ASD, and 19.3% with EmD as the sole diagnosis. An additional 21.7% of CYP had co-occurrent ADHD and ASD, 3.6% both ADHD and EmD, and 3% both ASD and EmD. Less than 1% received all three diagnoses.

**Table 1:** Socio-demographic and clinical characteristics

	Parent_only responses *N* = 795	YP_only responses *N* = 762	Both responses *N* = 946	Any responder *N* = 2503 (46.5%)	Non-responders *N* = 2883 (53.5%)	Whole sample *N* = 5386
Age at lockdown (years) Mean (SD)	13.2 (3.2)	13.5 (3.3)	12 .9 (3.2)	13.2 (3.2)	13.6 (3.2)	13.4 (3.2)
School age groups *N* (%)						
Primary school	262 (32.9%)	231 (30.3%)	361 (38.1%)	854 (34.1%)	812 (28.1%)	1666 (30.9%)
Secondary school	304 (38.2%)	266 (34.9%)	348 (36.7%)	918 (36.6%)	1130 (55.1%)	2048 (38%)
College	229 (28.8%)	265 (34.7%)	237 (25%)	731 (29.2%)	941 (32.6%)	1672 (31%)
Sex *N* (%)^a^						
Male	472 (59.5%)	399 (52.5%)	504 (53.5%)	1375 (55.1%)	1681 (58.5%)	3056 (56.9%)
Female	321 (40.4%)	361 (47.5%)	438 (46.5%)	1120 (44.8%)	1188 (41.4%)	2308 (43%)
Ethnicity *N* (%)						
White	440 (55.3%)	315 (41.3%)	488 (51.5%)	1243 (49.6%)	1239 (42.9%)	2482 (46%)
Black	123 (15.4%)	178 (23.3%)	170 (17.9%)	471 (18.8%)	680 (23.5%)	1151 (21.3%)
Asian	10 (1.2%)	21 (2.7%)	15 (1.5%)	46 (1.8%)	68 (2.36%)	114 (2.1%)
Mixed	85 (10.6%)	76 (9.9%)	93 (9.8%)	254 (10.1%)	349 (12.1%)	603 (11.2%)
Other	12 (1.5%)	19 (2.4%)	12 (1.2%)	43 (1.72%)	45 (1.56%)	88 (1.6%)
Non-stated	125 (15.7%)	153 (20%)	168 (17.7%)	446 (17.8%)	502 (17.4%)	948 (17.6%)
Neighborhood deprivation *N* (%)^a^						
Least deprived	193 (24.9%)	131 (17.5%)	224 (24.5%)	548 (22.5%)	500 (17.8%)	1048 (20%)
2nd least deprived	186 (24%)	135 (18.1%)	174 (19%)	495 (20.3%)	553 (19.7%)	1048 (20%)
3rd least deprived	130 (16.8%)	150 (20.1%)	176 (19.2%)	456 (18.7%)	585 (20.8%)	1041 (19.8%)
2nd most deprived	124 (16%)	161 (21.6%)	169 (18.4%)	454 (18.6%)	600 (21.3%)	1054 (20.1%)
Most deprived	141 (18.2%)	168 (22.5%)	171 (18.7%)	480 (19.7%)	569 (20.2%)	1049 (20%)
Primary diagnosis *N* (%)						
ADHD	140 (17.6%)	105 (13.7%)	149 (15.7%)	394 (15.7%)	491 (17%)	885 (16.43%)
ASD	118 (14.8%)	120 (15.7%)	126 (13.3%)	364(14.5%)	384 (13.3%)	748 (13.8%)
CD/ODD	18 (2.2%)	18 (2.3%)	12 (1.2%)	48 (1.9%)	75 (2.6%)	123 (2.2%)
Depressive disorders	21 (2.6%)	38 (4.9%)	30 (3.1%)	89 (3.5%)	109 (3.7%)	198 (3.6%)
Anxiety disorders	91 (11.4%)	82 (10.7%)	106 (3.1%)	279 (11.1%)	293 (10.1%)	572 (10.6%)
PTSD	5 (0.6%)	21 (2.6%)	12 (1.2%)	38 (1.5%)	70 (2.4%)	108 (2%)
ED	31 (3.9%)	29 (3.8%)	34 (3.5%)	94 (3.7%)	63 (2.1%)	157 (2.9%)
Psychosis	3 (0.3%)	6 (0.7%)	4 (0.4%)	13 (0.5%)	17 (0.5%)	30 (0.5%)
OCD	25 (3.1%)	17 (2.2%)	22 (2.3%)	64 (2.5%)	73 (2.5%)	137 (2.5%)
Non-psychiatric diagnosis	1 (0.1%)	0	3 (0.3%)	4 (0.1%)	1 (0.03%)	5 (0.09%)
Non specified	117 (14.7%)	108 (14.1%)	127 (13.4%)	352 (14%)	470 (16.3%)	822 (15.2%)
Other uncommon	16 (2%)	25 (3.2%)	22 (2.3%)	63 (2.5%)	91 (3.1%)	154 (2.8%)
3 main diagnoses (including comorbidities) *N* (%)						
ADHD	195 (35.3%)	157 (29.7%)	216 (33.1%)	568 (32.7%)	718 (36.9%)	1286 (35%)
ASD	215 (38.9%)	213 (40.3%)	266 (40.8%)	694 (40%)	678 (34.9%)	1372 (37.3%)
EmD^b^	142 (25.7%)	158 (29.9%)	170 (26%)	470 (27.1%)	545 (28%%)	1015 (27.6%)
ID *N* (%)	37 (4.6%)	40 (5.2%)	33 (3.49%)	110 (4.3%)	101 (3.5%)	211 (3.9%)

This table displays pre-Covid socio-demographic and clinical characteristics of the sampling frame, of the group that took part to the survey according to the main respondent (parent only, young person only or both), and in the group that did not respond to the survey

*ADHD* Attention Deficit Hyperactivity Disorder, *ASD* Autism Spectrum Disorder, *CD* Conduct Disorder, *ED* Eating Disorder, *EmD* Emotional Disorders, *ID* Intellectual Disability, *N* number, *OCD* Obsessive–Compulsive Disorder, *ODD* Oppositional Defiant Disorder, *PTSD* Post-Traumatic Stress Disorder, *SD* standard deviation, *YP* young person

^a^The following variables have missing data: sex (*N* = 22), neighborhood deprivation (*N* = 146)

^b^Emotional disorders include depressive disorders, anxiety disorders, PTSD, and OCD

### Covid impact on family life, education and CYP’s mental health

At the time of the survey, a minority of parents reported that a family member had been hospitalized (1.5%) or passed away (1.5%) due to Covid. However, 12% were furloughed or dismissed; 45% reported some level of financial difficulties; and 49% experienced housing inadequacy. A higher proportion of CYP attended school in-person as compared to national estimates (42% vs 17.5%) (Children’s Commissioner—Debriefing, December 2020). CYP’s emotional and behavioral symptoms worsened in 47% and 40.5% of cases, respectively, and 72.5% of parents stated that CAMHS was meeting their child’s needs.

### Children with neurodevelopmental disorders were the most affected

**Fig. 1:**
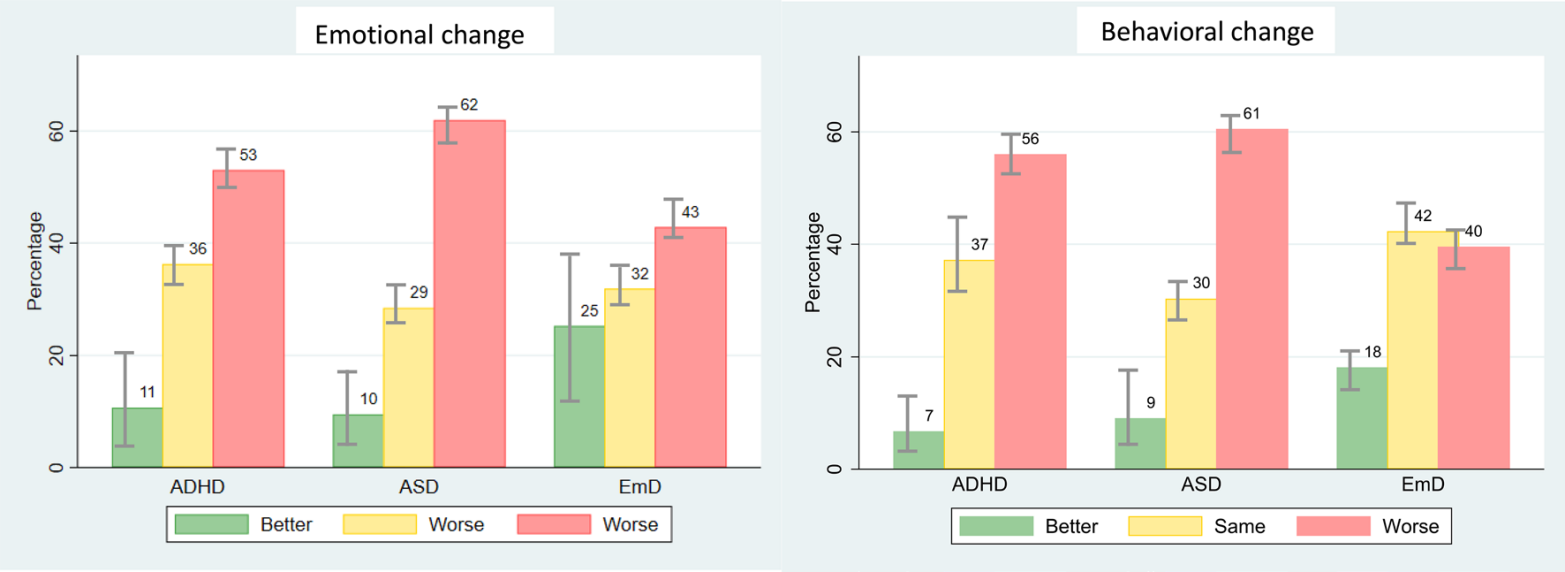
Emotional and behavioral changes in the three main diagnostic groups. This figure displays the proportion of CYP with better or worse emotional and behavioral change, or no change, as compared to before the pandemic. Emotional and behavioral change were derived from individual Likert scale questions on symptom change but, for visualization purposes, the five categories were collapsed into three. For all outcomes, higher scores reflected symptom worsening. *ADHD* Attention Deficit Hyperactivity Disorder, *ASD* Autism Spectrum Disorder, *CYP* children and young people, *EmD* Emotional Disorders

Within our clinical population, we observed significant differences among diagnostic groups for all four parent-rated outcomes (Table 2). Post hoc *t*-tests showed that ASD and EmD were significantly associated with more emotional difficulties than ADHD (*t*(891) = − 4.431, *p* < 0.001 and *t*(590) = − 3.254, *p* = 0.001); and both ADHD and ASD with more behavioral difficulties than EmD (*t*(591) = 5.618, *p* = 0.001 and *t*(663) = 6.527, *p* = 0.001). Greater emotional deterioration was observed in CYP with ASD, as compared to those with EmD (*t*(664) = 4.670, *p* < 0.001) or ADHD (*t*(893) = − 3.041, *p* = 0.002); and in CYP with ADHD as compared to those with EmD (*t*(591) = 2.592, *p* = 0.009). Similarly, greater behavioral deterioration was reported in CYP with ADHD or ASD, as compared to those with EmD (*t*(594) = 4.529, *p* < 0.001 and *t*(664) = 5.082, *p* < 0.001). Emotional and behavioral change in the three main diagnostic groups are displayed in Fig. 1. Significant differences among diagnostic groups were also observed for parent-rated child’s Covid-related worries (Table 2), which were higher in ASD than in ADHD (*t*(889) = − 2.125, *p* = 0.033). Considering contextual factors, parents of CYP with ADHD or ASD reported significantly greater housing inadequacy (*t*(551) = 3.305, *p* = 0.001 and *t*(626) = 3.381, *p* = 0.001); lack of family support (*t*(532) = 3.519, *p* < 0.001 and *t*(603) = 4.062, *p* < 0.001); and difficulties with social distancing (*t*(538) = 4.239, *p* < 0.001 and *t*(608) = 4.949, *p* < 0.001). Finally, CYP with ADHD spent more time outside than those with ASD (*t*(827) = − 4.428, *p* < 0.001) or EmD (*t*(551) = − 2.629, *p* = 0.008) (Table 2). We did not observe significant group differences in child relationships; parent mental health; parental concerns; challenges with education; and perceived mental health care (Table 2). Of note, the observed variation in degrees of freedom in the comparative analyses is due to missing survey responses (see “Discussion”).

**Table 2:** Comparison of main and aggregated parents’ survey responses among the three main diagnostic groups

	Whole sample	Main diagnostic groups				
	Parent responses *N* = 1741	ADHD *N* = 632	ASD *N* = 764	EmD^a^ *N* = 336	*F* (*p*-value)	Post hoc *t*-test (*p*-value)
Total emotional score Mean (SD)	17.62 (5.09)	17.04 (4.81)	18.53 (5.18)	18.47 (5.17)	**F(2, 1071) = 10.87 (< 0.001)**	
	*N* = 1074	*N* = 411	*N* = 482	*N* = 181	ADHD vs. ASD	**t(891) = − 4.431 (< 0.001)**
					ADHD vs. EmD	**t(590) = − 3.254 (0.001)**
Total behavioral score Mean (SD)	12.00 (4.00)	12.59 (3.83)	12.87 (3.89)	10.71 (3.57)	**F(2, 1073) = 22.03 (< 0.001)**	
	*N* = 1076	*N* = 411	*N* = 483	*N* = 182	ADHD vs. EmD	**t(591) = 5.618 (0.001)**
					ASD vs. EmD	**t(663) = 6.527 (0.001)**
Emotional change Mean (SD)	3.50 (1.04)	3.52 (0.92)	3.72 (1.01)	3.29 (1.21)	**F(2,1074) = 12.84 (< 0.001)**	
	*N* = 1077	*N* = 411	*N* = 484	*N* = 182	ADHD vs. ASD	**t(893) = − 3.041 (0.002)**
					ADHD vs. EmD	**t(591) = 2.592 (0.009)**
					ASD vs. EmD	**t(664) = 4.670 (< 0.001)**
Behavioral change Mean (SD)	3.58 (1.02)	3.69 (0.92)	3.76 (1.03)	3.30 (1.05)	**F(2,1077) = 14.47 (< 0.001)**	
	*N* = 1080	*N* = 414	*N* = 484	*N* = 182	ADHD vs. EmD	**t(594) = 4.529 (< 0.001)**
					ASD vs. EmD	**t(664) = 5.082 (< 0.001)**
Covid-19-related worries Mean (SD)	4.90 (2.42)	4.90 (2.40)	5.26 (2.59)	4.84 (2.21)	**F(2, 1070) = 3.17 (0.042)**	
	*N* = 1073	*N* = 411	*N* = 480	*N* = 182	ADHD vs. ASD	**t(889) = − 2.125 (0.033)**
Poor quality of child’s relationships Mean (SD)	13.76 (5.84)	13.89 (5.64)	13.97 (6.38)	13.63 (5.41)	F(2, 1029) = 0.22 (0.806)	
	*N* = 1032	*N* = 392	*N* = 464	*N* = 176		
Housing inadequacy Mean (SD)	3.03 (1.22)	3.12 (1.33)	3.09 (1.21)	2.74 (1.06)	**F(2, 1003) = 6.35 (0.001)**	
	*N* = 1006	*N* = 378	*N* = 453	*N* = 175	ADHD vs. EmD	**t(551) = 3.305 (0.001)**
					ASD vs. EmD	**t(626) = 3.381 (0.001)**
Poor parental mental health Mean (SD)	28.68 (7.57)	28.66 (7.48)	29.40 (7.63)	27.83(7.59)	F(2, 982) = 2.88 (0.056)	
	*N* = 985	*N* = 369	*N* = 444	*N* = 172		
Lack of family support Mean (SD)	7.28 (2.29)	7.46 (2.33)	7.54 (2.27)	6.71 (2.23)	**F(2, 966) = 8.58 (< 0.001)**	
	*N* = 969	*N* = 364	*N* = 435	*N* = 170	ADHD vs. EmD	**t(532) = 3.519 (< 0.001)**
					ASD vs. EmD	**t(603) = 4.062 (< 0.001)**
Parental concerns Mean (SD)	4.46 (1.96)	4.55 (2.01)	4.55 (1.95)	4.32 (1.81)	F(2, 969) = 0.98 (0.375)	
	*N* = 972	*N* = 367	*N* = 434	*N* = 171		
Challenges with education Mean (SD)	6.59 (2.31)	6.72 (2.29)	6.85 (2.32)	6.49 (2.39)	F(2, 1016) = 1.53 (0.217)	
	*N* = 1019	*N* = 387	*N* = 457	*N* = 175		
Perceived inadequacy of child’s MH care Mean (SD)	8.16 (1.85)	7.99 (1.84)	8.22 (1.95)	8.15 (1.66)	F(2, 1023) = 1.65 (0.192)	
	*N* = 1026	*N* = 387	*N* = 463	*N* = 176		
Limited outdoor time Mean (SD)	2.04 (0.86)	2.81 (0.96)	3.10 (0.90)	3.04 (0.90)	**F(2,1000) = 10.34 (< 0.001)**	
	*N* = 1003	*N* = 379	*N* = 450	*N* = 174	ADHD vs. ASD	**t(827) = − 4.428 (< 0.001)**
					ADHD vs. EmD	**t(551) = − 2.629 (0.008)**
Difficulty with ‘social distancing’ Mean (SD)	2.96 (0.92)	2.10 (0.88)	2.16 (0.91)	1.77 (0.73)	**F(2,975) = 12.48 (< 0.001)**	
	*N* = 978	*N* = 329	*N* = 390	*N* = 259	ADHD vs. EmD	**t(538) = 4.239 (< 0.001)**
					ASD vs. EmD	**t(608) = 4.949 (< 0.001)**

Significant results are in bold

This table reports average scores for the main and aggregated parents’ survey responses, and the results of the one-way ANOVAs and post hoc *t*-tests comparing the three main diagnostic groups. Please note that degrees of freedom vary due to missing data. Number of available observations is reported

*ADHD* Attention Deficit Hyperactivity Disorder, *ASD* Autism Spectrum Disorder, *EmD* Emotional Disorders, *MH* mental health, *SD* standard deviation

^a^Emotional disorders include depressive disorders, anxiety disorders, Post-Traumatic Stress Disorder, and Obsessive–Compulsive Disorder

### Poor parental mental health and educational challenges were the most important associated factors for worse outcome

Unadjusted and adjusted regressions identified pre-Covid socio-demographic and Covid-related contextual characteristics and co-occurrent disorders associated with worse mental health outcomes in the whole sample and in the three main diagnostic groups (Tables S3–4 and S5–8). For visualization purposes, Table 3 displays the significant results of the fully adjusted models.

**Table 3:**
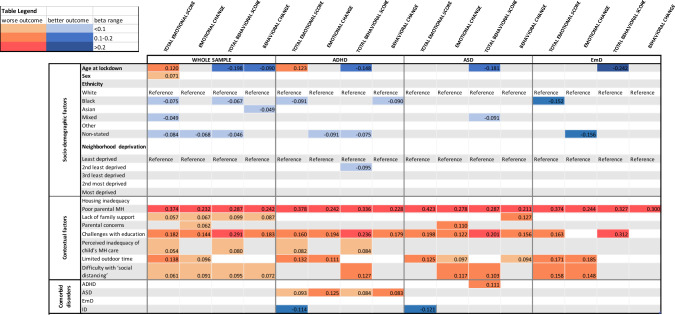
Heat map

For visualization purposes, this table displays the results of the fully adjusted regression models that were significant at *p* < 0.05 in the whole sample and the three main diagnostic groups. Results are displayed as standardized beta for ease of interpretation. Those in orange are the factors associated with worse mental health outcomes, those in blue with better mental health outcomes. The darker the color the stronger the association observed. Full regression results are presented in Tables S5–8

Considering pre-Covid socio-demographic characteristics in the *whole sample*, the fully adjusted models showed that age was an important risk factor, but its effect varied according to the outcome considered: being older was associated with more emotional difficulties (*β* = 0.190 [95% CI 0.114 0.267]), whilst being younger with more behavioral problems (− 2.248 [− 0.307 − 0.190]) and greater behavioral deterioration (− 0.029 [− 0.046 − 0.012]). Being female was also associated with more emotional difficulties (0.733 [0.255 1.211]). Conversely, parents of Black and Mixed ethnicity CYP reported lower levels of emotional difficulties (− 1.043 [− 1.721 − 0.365] and − 0.816 [− 1.587 − 0.045]); and those of Black and Asian ethnicity lower levels of behavioral difficulties (− 0.738 [− 1.253 − 0.223]) and behavioral deterioration (− 0.398 [− 0.796 0.001]), respectively. Among contextual variables, poor parental mental health and educational challenges were the most important associated factors for all outcomes, followed by lack of family support and difficulty with social distancing (Tables S5–8). Of note, the relationships between parent and child mental health held in the sensitivity analysis using child reports on their own mental health, except for emotional change (*p* = 0.08) (Supplementary results, 2.3). Finally, parental concerns regarding housing/finances were associated with greater emotional deterioration (0.034 [0.001 0.067]); perceived inadequacy of mental health care with more emotional (0.151 [0.027 0.276]) and behavioral difficulties (0.177 [0.082 0.272]); and limited outdoor time with more emotional difficulties (0.760 [0.501 1.018]) and emotional deterioration (0.110 [0.051 0.170]). We did not observe significant associations with neighborhood deprivation and housing inadequacy.

The following associations held in all the *three diagnostic groups* (Tables S5–8): younger age was associated with more behavioral problems; poor parental mental health and educational challenges with all outcomes (apart from emotional and behavioral deterioration in EmD); and limited outdoor time with total emotional difficulties and deterioration. We found that only in the *ADHD group*, Black ethnicity was associated with improvement in behavioral change ratings (− 0.299 [− 0.445 − 0.013]); living in the second least deprived areas with lower levels of behavioral difficulties (− 0.907 [− 1.737 − 0.076]); and co-occurring ASD with worse emotional and behavioral outcomes (Tables S5–8). Co-occurring ID was associated with fewer emotional difficulties in both the ADHD and ASD groups (− 3.040 [− 5.014 − 1.066] and − 2.141 [− 3.681 − 0.600]). Only in the *ASD group*, we observed that Mixed ethnicity was associated with lower levels of behavioral difficulties (− 1.379 [− 2.542 − 0.215]) and limited outdoor time with greater behavioral deterioration (0.105 [0.001 0.209]). Co-occurring ADHD was associated with worse behavioral problems (0.888 [0.223 1.553]). Finally, we did not observe *EmD group*-specific associations (Tables S5–8). Of note, the second sensitivity analysis showed that most of the observed differences among diagnostic groups were independent from contextual and socio-demographic factors (Supplementary results, 2.3).

### Educational challenges were associated with worse mental health

**Fig. 2:**
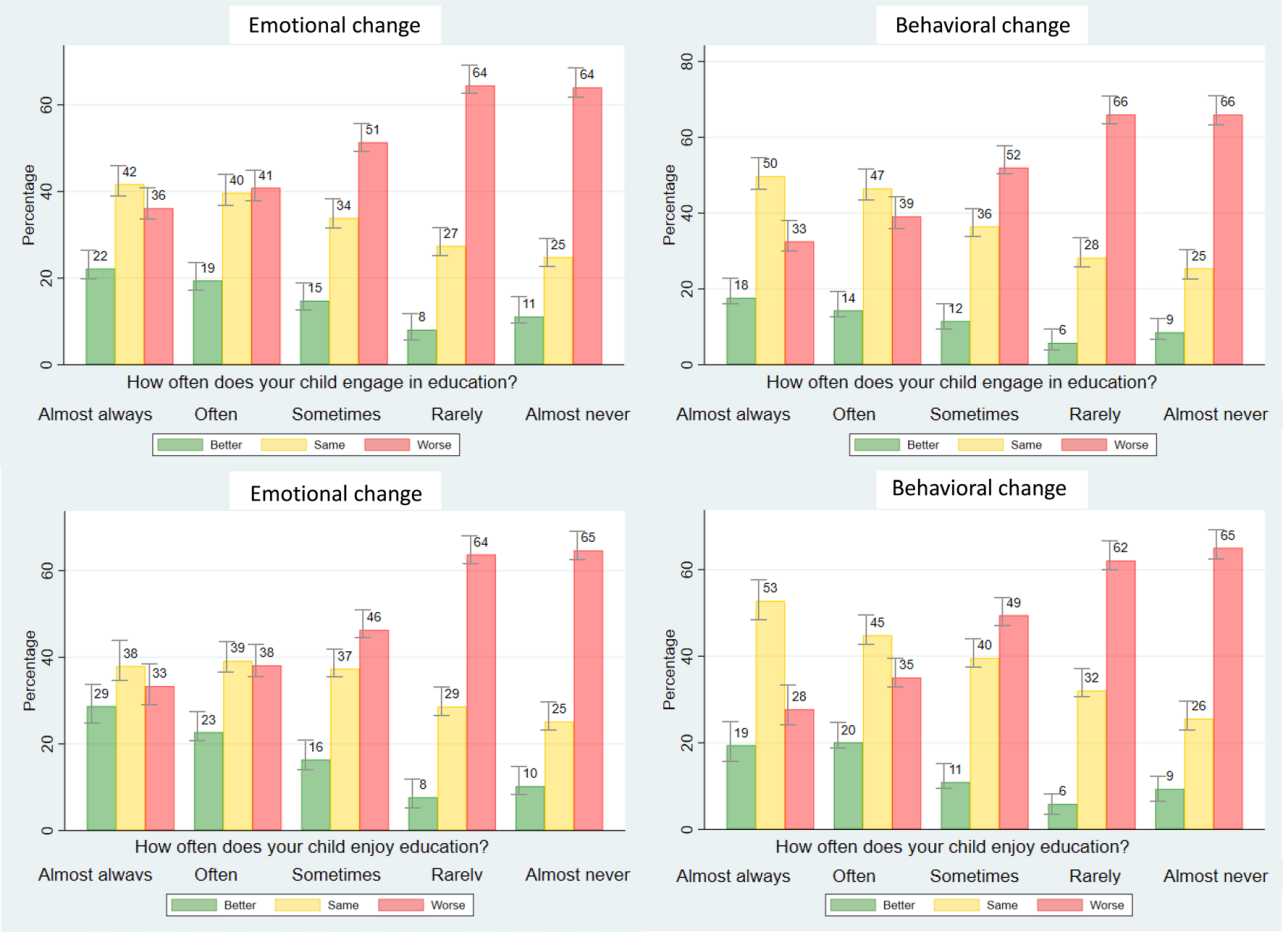
Emotional and behavioral change according to education enjoyment and engagement. This figure displays the proportion of CYP in the whole sample with parent responses that showed better or worse emotional and behavioral change, or no change, as compared to before the pandemic according to the reported frequency of education enjoyment or engagement. Emotional and behavioral change were derived from individual Likert scale questions on symptom change but, for visualization purposes, the five categories were collapsed into three. *ADHD* Attention Deficit Hyperactivity Disorder, *ASD* Autism Spectrum Disorder, *CYP* children and young people, *EmD* Emotional Disorders

Full analyses on education are reported in Supplementary results (Sect.  2.4). In brief, considering CYP who attended education remotely, those with ASD had lower educational enjoyment than those with EmD (*t*(322) = 2.664, *p* = 0.008), and both those with ASD and ADHD had lower educational engagement than those with EmD (*t*(321) = 3.263, *p* = 0.001 and *t*(300) = 2.058, *p* = 0.040). Further, CYP with ASD and ADHD enjoyed remote education less than in-person (*t*(355) = − 2.38, *p* < 0.05 and *t*(425) = − 2.83, *p* < 0.01). Finally, we observed an inverse significant relationship between education engagement/enjoyment and emotional/behavioral deterioration in the whole sample and the three diagnostic groups (except education engagement/emotional change for EmD) (Fig. 2 and Table S10).

## Discussion

Our results showed that among CYP receiving support from mental health services pre-pandemic, those with ASD or ADHD were the most affected during social restrictions. However, irrespective of pre-pandemic diagnosis, the negative effects on family life, education and mental health have been extensive; and poor parental mental health and challenges with education were most strongly associated with worse emotional and behavioral outcomes.

Results that may be most informative for care pathway and resource planning are those on the differences among diagnostic groups, showing that CYP with ADHD or ASD were the most adversely affected by restrictions. Previous studies mainly focused on small samples with a single or mixed diagnosis and did not provide comparisons. These studies reported either a worsening of pre-pandemic symptomatology or development of new symptoms [12, 24, 25], especially in those with more severe illness pre-pandemic [17, 26]. A prior study in a small mixed sample showed that behavioral problems were more evident in CYP with ADHD and worsening prosocial behavior in those with ASD [10]. We observed that CYP with ADHD had similar behavioral but less emotional deterioration than those with ASD, and that both had more difficulties with social distancing and housing adequacy during lockdown as compared to those with EmD. Their coping difficulties may be partially related to different underlying mechanisms as, for instance, CYP with ADHD struggled the most with spending time at home, which may be related to impulsivity and hyperactivity; whilst those with ASD had more Covid-related worries and spent less time outdoor even for allowed activities. Finally, we observed that CYP with EmD were less adversely affected, and a greater proportion showed an improvement in emotional symptoms. There are different potential explanations for these findings, as CYP with EmD may be more resilient and/or may thrive more in the home environment, where they are less exposed to the school-associated stress that often occurs in EmD [18, 27].

Our findings suggest that both pre-Covid socio-demographic and Covid-related contextual factors play a role when considering the impact of the pandemic on CYP. In agreement with previous studies, we found that behavioral problems were associated with younger age and emotional problems with older age and female sex [9, 10, 28, 29]. Unexpectedly, parents of Black and ethnic minorities reported lower levels of emotional and behavioral difficulties, which contrasts with the growing concern on the disproportionate impact of the pandemic on minority ethnic groups [30, 31]. This may be due to a selection bias and reflect the characteristics of families that responded to the survey. Alternatively, as these communities are highly represented in our catchment area, it is possible that the reliance on a large family/community network could buffer the effects of the pandemic [30]. Similarly, neighborhood deprivation did not appear to be a significant associated factor, in contrast to previous studies [17, 26]. This may be due to residual confounding when controlling for an area-based measure of socio-economic deprivation [32], or to the fact that our catchment area mainly included very deprived boroughs, thus we may have been less powered to detect differences between higher and lower income neighborhoods. Regarding contextual factors, poor parental mental health was among the most important. The association between children’s and parents’ mental health has long been recognized and linked to both the direct effects of problematic parenting and indirect factors, such as family conflict and socio-economic deprivation [33]. Prior studies also highlighted the reciprocal interaction between children’s and parents’ mental health during the pandemic, especially when the child had additional needs [26, 34]. For instance, a child’s externalizing behavior was associated with increased parental stress [35] and parental low mood was associated with worse child’s ADHD symptoms [24]. In sum, the worsening of CYP’s symptoms may increase strain on parents, at a time when they may struggle themselves due to their own mental health, financial concerns, and lack of social support, and may be less able to respond to their children’s increased needs [17].

Finally, results on the impact of changes in education may be important for policy around education provision and for teachers. We observed that CYP with neurodevelopmental disorders, especially those with ASD, struggled more with remote education than those with EmD. Similarly, a prior study reported that CYP with ADHD had more remote learning difficulties than those without ADHD [19]. These results may reflect their reliance on the routine and physical environment provided by school. Further, low education enjoyment/engagement were associated with greater emotional and behavioral deterioration. Although these associations are based on cross-sectional data, and may potentially reflect a reciprocal interaction, they suggest that CYP’s experience of education is strongly related to their mental health outcomes.

This study has several strengths. It is one of the largest surveys internationally of CYP with pre-existing mental disorders with available pre-Covid clinicodemographic information; it is based on a well-characterized and ethnically diverse clinical population; and considers a comprehensive range of pandemic-related variables. All these factors enhance the generalizability of findings. They also allowed us to obtain relatively precise estimates; provide clinicodemographic characteristics of both responders and non-responders; and include ethnic groups that may be otherwise hard to reach. However, limitations should also be considered. Considering the study design, this is the first (cross-sectional) report of our longitudinal study, and our results need to be complemented by longitudinal data to draw more robust conclusions on the relationship between exposures and outcomes. It also focuses on the initial period of the pandemic but, as its impact is evolving over time, our results need to be complemented by the data we collected at following time points, for which they represent a valuable baseline reference. Considering the sample, we surveyed a large clinical population, but we did not have a normative sample; thus, further studies should investigate whether children with pre-existing mental disorders fared worse than those in the general population. Only 32% of caregivers/CYP completed the survey and some submitted incomplete questionnaires. We noted that questions with lower response rates were those on parent circumstances and respect of social distancing measures (Table 2). Thus, findings may not be completely generalizable to the whole clinical population served by out Trust; however, results suggest that responders and non-responders had similar baseline clinicodemographic characteristics and the inclusion of all available data, instead of completed surveys only, reduced potential bias. We were unable to control for baseline severity at the start of the pandemic as many CYP did not have an up-to-date structured measure of symptoms or function recorded at that time. We considered the most common diagnoses, but those that received less attention in the Covid literature need also to be investigated (e.g., eating disorders and psychosis). Considering the outcomes, we measured symptom-based outcomes, but others can be considered in future studies (e.g., the increasing rates of A&E presentations or self-harm in CYP). The survey did not include validated measures of symptom severity; however, questions were based on the standard CRISIS questionnaire, which has been used in many pandemic-related studies, and we used factor analysis to confirm the underlying structure. We asked parents to rate symptom change as compared to before the pandemic, as the survey was developed *at hoc* to investigate the effects of the pandemic and, thus, we did not have pre-pandemic scores for comparison. Finally, we considered a broad range of factors potentially affecting mental health outcomes in a large sample, and tested the robustness of findings in sensitivity analyses, but did not correct for multiple comparisons. This was due to the exploratory nature of the analysis and the lack of well-known robust associates of worse mental health outcome in the literature of CYP with mental disorders. Nevertheless, our findings may provide a reference for future studies aiming at the identification of the most relevant risk factors in this vulnerable population.

In conclusion, our findings suggest that mental disorders, especially ASD and ADHD, may negatively affect CYP’s resilience and flexibility to adapt to rapid changes, especially when they experience additional challenges in their support network. Knowledge of the burden that social restrictions placed on CYP and families is important to guide clinical recognition, resourcing, and policy. Finally, increasing the availability of evidence-based treatments that reduce parental stress and support education may help mitigate the mental health impact of future pandemics.

### Supplementary Information

Below is the link to the electronic supplementary material.

Supplementary file1 (PDF 847 KB)

Supplementary file2 (PDF 1332 KB)

## Data Availability

The data accessed by CRIS remain within an NHS firewall and governance is provided by a patient-led oversight committee. Access to data is restricted to honorary or substantive employees of the South London and Maudsley NHS Foundation Trust and governed by a local oversight committee who review and approve applications to extract and analyze data for research. Subject to these conditions, data access is encouraged and those interested should contact RS (robert.stewart@kcl.ac.uk), CRIS academic lead.
