# Supplementary material for: Emotional and behavioral outcomes among youths with mental disorders during the first Covid lockdown and school closures in England: a large clinical population study using health care record integrated surveys
Source: Soc Psychiatry Psychiatr Epidemiol. 2023 Jun 23;59(1):175–86. doi: 10.1007/s00127-023-02517-w (PMC10799796; doi:10.1007/s00127-023-02517-w)
Supplement: Supplementary file 1 — Supplementary file1 (PDF 847 KB) [file 127_2023_2517_MOESM1_ESM.pdf]

APPENDIX

Emotional and behavioral outcomes among youths with mental disorders during the first Covid lockdown and school closures in England: a large clinical population study using health care record integrated surveys.

European Child and Adolescent Psychiatry.

Parlatini V. [1], Frangou L. [1]*, Zhang S. [1]*, Epstein S. [1], Morris A. [1], Grant C. [1,2], Zalewski L. [3,4], Jewell A. [4], Velupillai S. [3], Simonoff E.[1,4]¶, Downs J. [1,4]¶

* ¶ These authors equally contributed to the study

[1] Department of Child and Adolescent Psychiatry, Institute of Psychiatry, Psychology and Neuroscience, Kings College London, London, UK

[2] Department of Epidemiology & Public Health, University College London, London, UK

[3] Department of Biostatistics & Health Informatics, Institute of Psychiatry, Psychology and Neuroscience, King's College London, London, UK

[4] National Institute for Health Research (NIHR) Biomedical Research Centre, South London and Maudsley NHS Foundation Trust, London, UK

Corresponding author: valeria.parlatini@kcl.ac.uk

CYPHER survey – 1st wave

The following tables report the Maudsley Child and Young People Health & Experience Research (CYPHER) survey questions for parents and young people; proportion of responses provided; and correlations between average matched parent and young people responses. Please note, in this study we did not analyze open questions, thus we did not include these in the tables.

Questions included in composite scores are color-coded as follows:

Outcomes
Total emotional score: yellow
Total behavioural score: light blue
Emotional and behavioural changes: orange

Other composite scores
Covid-related worries: light green
Parent mental health: pink
Lack of family support: water green
Parent concerns: red
Housing inadequacy: purple
Challenges with education: green
Experience of child's mental health care: fuchsia
Quality of child's relationships: blue

Question	COVID-19 EXPOSURE AND PHYSICAL HEALTH	PARENT N=1741	YOUNG PEOPLE N=1708	r (p-value)*
Q2	**Have you been suspected of having Coronavirus/COVID-19 infection?**			
	No, not suspected	1605 (92.1%)		
	Yes, medical diagnosis, but no test	13 (0.7%)		
	Yes, waiting for test result	3 (0.1%)		
	Yes, a positive test result	9 (0.5%)		
	Yes, a negative test result	30 (1.7%)		
	I'd rather not say	7 (0.4%)		
	Missing	74 (4.2%)		
Q3	**Have any of the following happened to you because of Coronavirus/COVID-19? (check all that apply)**			
	Fallen ill physically	7 (0.4%)		
	Hospitalized	1 (0.06%)		
	Self- isolation with symptoms	4 (0.2%)		
	Self- isolation without symptoms (e.g., due to possible exposure)	10 (0.5%)		
	None of the above	3 (0.1%)		
	Missing	1716 (98.5%)		
Q4	**Has anyone in your family been diagnosed with Coronavirus/COVID-19? (check all that apply)**			
	Yes, member of household	23 (1.3%)		
	Yes, non-household member	78 (4.4%)		
	No	1567 (90%)		
	Missing	77 (4.4%)		
Q5	**Have any of the following happened to your family members because of Coronavirus/COVID-19? (check all that apply)**			
	Fallen ill physically	36 (2%)		
	Hospitalized	31 (1.7%)		
	Self- isolation with symptoms	42 (2.4%)		
	Self- isolation without symptoms (e.g., due to possible exposure)	21 (1.2%)		
	Passed away	28 (1.6%)		
	NA/Missing	1647 (94.6%)		
Q6	**Has your child been suspected of having Coronavirus/COVID-19 infection?**			
	No, not suspected	1621 (93.11%)		
	Yes, medical diagnosis, but no test	10 (0.5%)		
	Yes, waiting for test result	3 (0.1%)		
	Yes, a positive test result	2 (0.1%)		
	Yes, a negative test result	13 (0.7%)		
	Yes, I do not want to share their test result	2 (0.1%)		
	Missing	90 (5.1%)		
Q7	**Have any of the following happened to your child because of Coronavirus/COVID-19? (check all that apply)**			
	Fallen ill physically	3 (0.1%)		
	Hospitalized	1 (0.05%)		
	Put into self-isolation with symptoms	7 (0.4%)		
	Put into self-isolation without symptoms (e.g., due to possible exposure)	3 (0.1%)		
	NA/Missing	1730 (99.3%)		
Q8 P/Q1 YP	**Has your child reported changes in their sense of smell?**<br>**have you reported any changes in your sense of smell?**			
	No	1626 (93.3%)	1212 (70.96%)	
	Yes, possibly	15 (0.8%)	46 (2.6%)	
	Yes, definitely	10 (0.5%)	28 (1.6%)	
	Missing	90 (5.1%)	422 (24.7%)	

Question	CHILD'S SYMPTOMS (PAST TWO WEEKS)	PARENT N=1741	YOUNG PERSON N=1708	r (p-value)*
Q9 P/Q2 YP	**how worried was your child generally?** **how worried were you generally?**			0.472 (<0.001)
	Not worried at all	357 (20.5%)	257 (15%)	
	Slightly worried	411 (23.6%)	365 (21.3%)	
	Moderately worried	399 (22.9%)	344 (20.1%)	
	Very worried	256 (14.7%)	221 (12.9%)	
	Extremely worried	130 (7.4%)	99 (5.8%)	
	Missing	188 (10.8%)	422 (24.7%)	
Q10 P/Q3 YP	**how happy versus sad was your child?** **how happy versus sad were you?**			0.430 (<0.001)
	Very sad/depressed/unhappy	144 (8.2%)	147 (8.6%)	
	Moderately sad/depressed/unhappy	484 (27.8%)	388 (22.7%)	
	Neutral	526 (30.2%)	493 (28.72%)	
	Moderately happy/cheerful	291 (16.7%)	190 (11.1%)	
	Very happy/cheerful	100 (5.7%)	68 (3.9%)	
	Missing	196 (11.2%)	422 (24.7%)	
Q11 P/Q4 YP	**how much has your child been able to enjoy his/her usual activities?** **how much have you been able to enjoy your usual activities?**			0.386 (<0.001)
	Not at all	295 (16.9%)	245 (14.3%)	
	Slightly	522 (29.9%)	442 (25.8%)	
	Moderately	508 (29.1%)	385 (22.5%)	
	Very much	181 (10.4%)	142 (8.3%)	
	A lot	45 (2.5%)	73 (4.2%)	
	Missing	190 (10.9%)	421 (24.6%)	
Q12 P/ Q5 YP	**how relaxed versus anxious was your child?** **how relaxed versus anxious were you?**			0.401 (<0.001)
	Very relaxed/calm	109 (6.2%)	107 (6.2%)	
	Moderately relaxed/calm	296 (17%)	215 (12.5%)	
	Neutral	342 (19.6%)	352(20.6%)	
	Moderately nervous/anxious	556 (31.9%)	424 (24.8%)	
	Very nervous/anxious	249 (14.3%)	187(10.9%)	
	Missing	189 (10.8%)	423 (24.7%)	
Q13 P/ Q6 YP	**how well has your child been sleeping?** **how well have you been sleeping?**			0.347 (<0.001)
	Sleeping much more than usual	141 (8.1%)	114 (6.6%)	
	Sleeping a little more than usual	194 (11.1%)	198 (11.5%)	
	Sleeping about the same	555 (31.8%)	397 (23.2%)	
	Sleeping a little less than usual	400 (22.9%)	337 (19.7%)	
	Sleeping a lot less than usual	263 (15.1%)	241 (14.1%)	
	Missing	188 (10.8%)	421 (24.6%)	
Q14 P/ Q7 YP	**how fidgety or restless was your child?** **how fidgety or restless were you?**			0.495 (<0.001)
	Not restless at all	247 (14.9%)	190 (11.1%)	
	Slightly restless	316 (18.1%)	282 (16.5%)	
	Moderately restless	416 (23.8%)	353 (20.6%)	
	Very restless	374 (21.4%)	298 (17.4%)	
	Extremely restless	197 (11.3%)	160 (9.3%)	
	Missing	191 (10.9%)	425 (24.8%)	
Q15 P/ Q8 YP	**how fatigued or tired was your child?** **how fatigued or tired were you?**			0.420 (<0.001)
	Not fatigued or tired at all	411 (23.6%)	260 (15.2%)	
	Slightly fatigued or tired	395 (22.6%)	335 (19.6%)	
	Moderately fatigued or tired	411 (23.6%)	335 (19.6%)	
	Very fatigued or tired	254 (14.5%)	245 (14.3%)	
	Extremely fatigued or tired	82 (4.7%)	110 (6.4%)	
	Missing	188 (10.8%)	423 (24.7%)	

Q16 P/ Q9 YP	**for their age, how well has your child been able to concentrate or focus? how well were you able to concentrate or focus?**			0.420 (<0.001)
	Very focused/attentive	81 (4.6%)	68 (3.9%)	
	Moderately focused/attentive	268 (15.3%)	193 (3.9%)	
	Neutral	318 (18.2%)	329 (19.2%)	
	Moderately unfocused/distracted	485 (27.8%)	432 (25.2%)	
	Very unfocused/distracted	400 (22.9%)	263 (15.4%)	
	Missing	189 (10.8%)	423 (24.7%)	
Q17 P/ Q10 YP	**how irritable or easily angered has your child been? how irritable or easily angered have you been?**			0.525 (<0.001)
	Not irritable or easily angered at all	160 (9.1%)	164 (9.6%)	
	Slightly irritable or easily angered	353 (20.2%	293 (17.1%)	
	Moderately irritable or easily angered	356 (20.4%)	297 (17.3%)	
	Very irritable or easily angered	401 (23%)	357 (20.9%)	
	Extremely irritable or easily angered	284 (16.3%)	176 (10.3%)	
	Missing	187 (10.7%)	421 (24.6%)	
Q18 P/ Q11 YP	**how physically aggressive towards others has your child been? how physically aggressive towards others have you been?**			0.651 (<0.001)
	No aggression at all	578 (33.2%)	470 (27.5%)	
	A little aggression, once or twice and not severe	340 (19.5%)	299 (17.5%)	
	Some aggression but not severe	331 (19%)	277 (16.2%)	
	Some aggression, including hurting others	183 (10.5%)	129 (7.5%)	
	Frequent aggression, including hurting others	121 (6.9%)	51 (2.9%)	
	Missing	188 (10.8%)	482 (28.2%)	
Q19 P/ Q12 YP	**how physically aggressive have others been towards your child? how physically aggressive have others been towards you?**			0.386 (<0.001)
	No aggression at all	1093 (62.7%)	743 (43.5%)	
	A little aggression, once or twice and not severe	265 (15.2%)	285 (16.6%)	
	Some aggression but not severe	136 (7.8%)	145 (8.4%)	
	Some aggression, including hurting others	37 (2.1%)	35 (2%)	
	Frequent aggression, including hurting your child	17 (0.9%)	15 (0.8%)	
	Missing	193 (11%)	485 (28.4%)	
Q20 P/ Q13 YP	**how lonely has your child been? how lonely have you been?**			0.485 (<0.001)
	Not lonely at all	409 (23.4%)	385 (22.5%)	
	Slightly lonely	427 (24.5%)	305 (17.8%)	
	Moderately lonely	364 (20.9%)	266 (15.5%)	
	Very lonely	225 (12.9%)	171 (10%)	
	Extremely lonely	126 (7.24%)	98 (5.7%)	
	Missing	190 (10.9%)	483 (28.2%)	
Q21 P/ Q14 YP	**how worried has your child been about being infected? how worried have you been about being infected?**			0.579 (<0.001)
	Not at all	497 (28.5%)	401 (23.4%)	
	Slightly	430 (24.7%)	332 (19.4%)	
	Moderately	275 (15.8%)	203 (11.8%)	
	Very	208 (11.9%)	149 (8.7%)	
	Extremely	139 (7.9%)	139 (8.1%)	
	Missing	192(11%)	484 (28.3%)	
Q22 P/ Q15 YP	**how worried has your child been about friends or family being infected? how worried have you been about friends or family being infected?**			0.521 (<0.001)
	Not at all	411 (26.5%)	186 (10.8%)	
	Slightly	464 (29.9%)	291 (17%)	
	Moderately	286 (18.4&)	263 (15.4%)	
	Very	238 (15.3%)	255 (14.9%)	
	Extremely	149 (9.6%)	229 (13.4%)	
	Missing	193 (11%)	484 (28.3%)	

Q26 P/ Q18 YP	**Overall, how would you compare your child's emotions (such as worries, sadness/happiness) to what they were like before the COVID pandemic?**<br>**Overall, how would you compare your emotions (such as worries, sadness/happiness) to what they were like before the COVID pandemic?**			0.375 (<0.001)
	Much worse	333 (19.1%)	196 (11.4%)	
	A little worse	481 (27.6%)	409 (23.9%)	
	About the same	566 (32.5%)	411 (24%)	
	A little better	118 (6.7%)	146 (8.5%)	
	Much better	58 (3.3%)	64 (3.7%)	
	Missing	185 (10.6%)	482 (28.2%)	
Q27 P/ Q19 YP	**Overall, how would you compare your child's behaviour (such as fidgetiness, concentration, aggression) to what they were like before the COVID pandemic?**<br>**Overall, how would you compare your emotions (such as worries, sadness/happiness) to what they were like before the COVID pandemic?**			0.448 (<0.001)
	Much worse	285 (10.8%)	224 (13.1%)	
	A little worse	517 (29.7%)	426 (24.9%)	
	About the same	526 (30.2%)	426 (24.9%)	
	A little better	152 (8.7%)	102 (5.9%)	
	Much better	73 (4.1%)	47 (2.7%)	
	Missing	188 (10.8%)	483 (28.2%)	

Question	CHILD RELATIONSHIP CHANGES AND THEIR IMPACT (PAST TWO WEEKS)	PARENT N=1741	YOUNG PERSON N=1708	r (p-value)*
Q28	**how would you rate the quality of your relationship with your son/daughter?**			
	Very poor	34 (1.9%)		
	Fairly poor	67 (3.8%)		
	Mixed	410 (23.5%)		
	Fairly good	463 (26.5%)		
	Very good	563 (32.3%)		
	Missing	204 (11.7%)		
Q29	**has the quality of the relationships between your child and his/her siblings changed?**			
	A lot worse	109 (6.2%)		
	A little worse	311 (17.8%)		
	About the same	568 (32.6%)		
	A little better	186 (10.6%)		
	A lot better	129 (7.4%)		
	NA/Missing	438 (25.1%)		
Q30	**how stressful have changes in contacts with extended family or friends been for your child?**			
	Not at all	339 (19.4%)		
	Slightly	383 (22%)		
	Moderately	419 (24%)		
	Very	251 (14.4%)		
	Extremely	143 (8.2%)		
	Missing	206 (11.83%)		
Q31 P/ Q21 YP	**have these emotions and behaviours affected your child's relationship with you and your partner, for better or worse? have these emotions and behaviours affected your relationship with your parents, for better or worse?**			0.309 (<0.001)
	Much better	79 (4.5%)	95 (5.5%)	
	A little better	173 (9.9%)	158 (9.2%)	
	About the same	799 (45.8%)	612 (35.8%)	
	A little worse	356 (20.4%)	268 (15.6%)	
	Much worse	97 (5.5%)	74 (4.3%)	
	Missing	237 (13.6%)	501 (29.3%)	
Q32 P/ Q22 YP	**have these emotions and behaviours affected your child's relationship with their siblings, for better or worse? have these emotions and behaviours affected your relationship with your brothers/sisters, for better or worse?**			0.434 (<0.001)
	Much better	60 (3.4%)	67 (3.9%)	
	A little better	150 (8.6%)	110 (6.4%)	
	About the same	601 (34.5%)	491 (28.7%)	
	A little worse	321 (18.4%)	270 (15.8%)	
	Much worse	129 (7.4%)	107 (6.2%)	
	NA/Missing	480 (27.5%)	663 (38.8%)	
Q33 P/ Q23 YP	**have these emotions and behaviours affected your child's relationship with their friends, for better or worse? have these emotions and behaviours affected your relationship with friends, for better or worse?**			0.397 (<0.001)
	Much better	40 (2.3%)	72 (4.2%)	
	A little better	73 (4.1%)	90 (5.2%)	
	About the same	1009 (57.9%)	712 (41.6%)	
	A little worse	269 (15.4%)	241 (14.1%)	
	Much worse	100 (5.7%)	84 (4.9%)	
	Missing	250 (14.3%)	509 (29.8%)	

Question	EXPERIENCE OF MENTAL HEALTH CARE FOR YOUR CHILD	PARENT N=1741	YOUNG PERSON N=1708	r (p-value)*
Q34 P/ Q24 YP	**How has the quality of mental health care changed over this time?how has the quality of mental health care that you are receiving changed over this time?**			0.463 (<0.001)
	A lot worse	193 (11%)	142 (8.3%)	
	A little worse	340 (19.5%)	198 (11.5%)	
	About the same	789 (45.3%)	668 (39.1%)	
	A little better	103 (5.9%)	102 (5.9%)	
	A lot better	49 (2.8%)	48 (2.8%)	
	Missing	267 (15.3%)	550 (32.2%)	
Q35 P/ Q25 YP	**How has the quantity of mental health care changed over this time? how has the quantity of your mental health care changed over this time?**			0.330 (<0.001)
	A lot less	234 (13.4%)	173 (10.1%)	
	A little less	236 (13.5%)	170 (9.9%)	
	About the same	794 (45.6%)	642 (37.5%)	
	A little more	132 (7.5%)	128 (7.4%)	
	A lot more	72 (7.5%)	44 (2.5%)	
	Missing	273 (15.6%)	551 (32.2%)	
Q36 P/ Q26 YP	**How well is the mental health care you are currently receiving meeting the needs of you and your child? how well is the mental health care meeting your needs?**			0.644 (<0.001)
	Not meeting our needs at all	341 (19.5%)	204 (11.9%)	
	Slightly meeting our needs	255 (14.6%)	210 (12.3%)	
	Moderately meeting our needs	282 (16.2%)	249 (14.5%)	
	Meeting our needs well	266 (15.2%)	206 (12%)	
	Meeting our needs very well	94 (5.4%)	83 (4.8%)	
	NA/Missing	503 (28.8%)	756 (44.2%)	

Question	EDUCATION AND CHILD CARE	PARENT N=1741	YOUNG PERSON N=1708	r (p-value)*
Q37	**What level of education is your child regularly enrolled in (before the COVID-19 pandemic)?**			
	Not currently in education	55 (3.1%)		
	Pre-school	4 (0.2%)		
	Primary School	561 (32.2%)		
	Secondary school (up to GCSEs)	679 (39%)		
	Sixth for or college	128 (7.3%)		
	Is home schooled	52 (2.9%)		
	Missing	262 (15%)		
Q38	**How have your child's education arrangements changed (past 2 weeks)?**			
	No change from before COVID-19	328 (18.4%)		
	Attends physical school at least some days	254 (14.5%)		
	Only attending school remotely, on part-time basis	446 (25.6%)		
	Only attending school remotely, on full-time basis	346 (19.8%)		
	Missing	367 (21%)		
Q39	**To what extent has looking after your child at home made it difficult to do your paid work and/or domestic duties?**			
	Almost never	307 (17.6%)		
	Rarely	175 (10%)		
	Sometimes	382 (21.9%)		
	Often	316 (18.1%)		
	Almost always	284 (16.3%)		
	Missing	277 (15.9%)		
Q40 P/ Q27 YP	**How much is your child currently enjoying his/her education? how much are you currently enjoying your education?**			0.597 (<0.001)
	Almost never	363 (20.8%)	362 (21.1%)	
	Rarely	340 (19.5%)	232 (13.5%)	
	Sometimes	458 (26.3%)	344 (20.1%)	
	Often	194 (11.1%)	133 (7.7%)	
	Almost always	108 (6.2%)	102 (5.9%)	
	Missing	278 (15.9%)	535 (31.3%)	
Q41 P/ Q28 YP	**How much is your child currently engaging in his/her distance education? how much are you currently engaging in your distance education?**			0.679 (<0.001)
	Almost never	350 (20.1%)	263 (15.4%)	
	Rarely	259 (14.8%)	179 (10.4%)	
	Sometimes	373 (21.4%)	291 (17%)	
	Often	258 (14.8%)	228 (13.3%)	
	Almost always	216 (12.4%)	199 (11.6%)	
	Missing	285 (16.3%)	548 (32%)	

Question	WORK-FAMILY BALANCE AND HOME ENVIRONEMNT (PAST TWO WEEKS)	PARENT N=1741	YOUNG PERSON N=1708	r (p-value)*
Q42	**to what extent have you felt focused and productive in your work and/or domestic duties?**			
	Almost never	137 (7.8%)		
	Rarely	263 (15.1%)		
	Sometimes	579 (33.2%)		
	Often	311 (17.8%)		
	Almost always	156 (8.9%)		
	Missing	295 (16.9%)		
Q43	**to what extent has your family found ways of dealing with things that have been difficult?**			
	Almost never	44 (2.5%)		
	Rarely	109 (6.2%)		
	Sometimes	607 (34.8%)		
	Often	485 (27.8%)		
	Almost always	196 (11.2%)		
	Missing	300 (17.2%)		
Q44	**to what extent have people in your family actively supported each other when upset about any aspect of the crisis?**			
	Almost never	51 (2.9%)		
	Rarely	87 (5%)		
	Sometimes	359 (20.6%)		
	Often	509 (29.2%)		
	Almost always	437 (25.1%)		
	Missing	298 (17.1%)		
Q45	**to what extent have people in your family provided practical, emotional and/or financial support to people in other Countries struggling with the crisis?**			
	Almost never	589 (33.8%)		
	Rarely	236 (13.5%)		
	Sometimes	338 (19.4%)		
	Often	154 (8.8%)		
	Almost always	100 (5.7%)		
	Missing	324 (18.6%)		
Q47	**How would you describe your home accommodation?**			
	Very comfortable, plenty of personal space for everyone	736 (42.2%)		
	Fairly comfortable, but with some sharing and occasionally difficult to have personal space	386 (22.1%)		
	Somewhat problematic with a lot of sharing but usually able to find some personal space when needed	171 (9.8%)		
	Very problematic, with no personal space	149 (8.5%)		
	Missing	299 (17.1%)		
Q48	**Do you have access to a personal garden/outside space that your family can use at any time?**			
	Yes	1127 (64.7%)		
	No	314 (18%)		
	Missing	300 (17.2%)		

Question	CAREGIVER'S EMOTIONS/WORRIES (PAST TWO WEEKS)	PARENT N=1741	YOUNG PERSON N=1708	r (p-value)*
Q49	**How worried have you been generally?**			
	Not worried at all	99 (5.6%)		
	Slightly worried	420 (24.1%)		
	Moderately worried	462 (26.5%)		
	Very worried	294 (16.8%)		
	Extremely worried	142 (8.1%)		
	Missing	324 (18.6%)		
Q50	**How happy versus sad have you been?**			
	Very sad/depressed/unhappy	101 (5.8%)		
	Moderately sad/depressed/unhappy	419 (24%)		
	Neutral	521 (29.9%)		
	Moderately happy/cheerful	325 (18.6%)		
	Very happy/cheerful	51 (2.9%)		
	Missing	324 (18.6%)		
Q51	**How much have you been able to enjoy your usual activities?**			
	Not at all	345 (19.8%)		
	Slightly	535 (30.7%)		
	Moderately	422 (24.2%)		
	Very much	89 (5.1%)		
	A lot	24 (1.3%)		
	Missing	326 (18.7%)		
Q52	**How relaxed versus anxious have you been?**			
	Very relaxed/calm	61 (3.5)		
	Moderately relaxed/calm	294 (16.8)		
	Neutral	417 (23.9%)		
	Moderately nervous/anxious	515 (29.5%)		
	Very nervous/anxious	128 (7.3%)		
	Missing	326 (18.7%)		
Q53	**How fidgety or restless have you been?**			
	Not restless at all	344 (19.7%)		
	Slightly restless	459 (26.3%)		
	Moderately restless	398 (22.8%)		
	Very restless	165 (9.4%)		
	Extremely restless	47 (2.7%)		
	Missing	328 (18.8%)		
Q54	**How fatigued or tired have you been?**			
	Not fatigued or tired at all	141 (8.1%)		
	Slightly fatigued or tired	391 (22.4%)		
	Moderately fatigued or tired	343 (19.7%)		
	Very fatigued or tired	342 (11.3%)		
	Extremely fatigued or tired	198 (11.3%)		
	Missing	326 (18.7%)		

Q55	**how well have you been able to concentrate or focus?**			
	Very focused/attentive	163 (9.3%)		
	Moderately focused/attentive	439 (9.3%)		
	Neutral	375 (25.2%)		
	Moderately unfocused/distracted	350 (20.1%)		
	Very unfocused/distracted	88 (5%)		
	Missing	326 (18.7%)		
Q56	**How irritable or easily angered have you been?**			
	Not irritable or easily angered at all	336 (19.3%)		
	Slightly irritable or easily angered	545 (31.3%)		
	Moderately irritable or easily angered	333 (19.1%)		
	Very irritable or easily angered	146 (8.3%)		
	Extremely irritable or easily angered	53 (3%)		
	Missing	328 (18.8%)		
Q57	**How lonely have you been?**			
	Not lonely at all	547 (31.4%)		
	Slightly lonely	393 (22.5%)		
	Moderately lonely	253 (14.5%)		
	Very lonely	146 (8.3%)		
	Extremely lonely	76 (4.3%)		
	Missing	326 (18.7%)		
Q58	**How worried were you that you or someone in your family would become infected?**			
	Not at all	161 (9.2%)		
	Slightly	447 (25.6%)		
	Moderately	331 (19%)		
	Very	297 (17%)		
	Extremely	179 (10.2%)		
	Missing	326 (18.7%)		

Question	CAREGIVER'S RELATIONSHIPS (PAST TWO WEEKS)	PARENT N=1741	YOUNG PERSON N=1708	r (p-value)*
Q61	**how would you rate the quality of your relationship with your partner?**			
	Very poor	31 (1.7%)		
	Not so good	56 (3.2%)		
	Mixed	189 (10.8%)		
	Quite good	242 (13.9%)		
	Very good	381 (21.88%)		
	NA/Missing	842 (48.3%)		
Q62	**how has the quality of your relationship with your partner since COVID-19?**			
	Much better	89 (5.1%)		
	A little better	126 (7.2%)		
	About the same	542 (31.1%)		
	A little worse	120 (6.8%)		
	Much worse	19 (1%)		
	Missing	845 (48.5%)		
Q63	**how would you rate the quality of the support you are receiving from your extended family and/or friends?**			
	Very unhelpful	102 (5.8%)		
	Unhelpful	112 (6.4%)		
	Mixed	502 (28.8%)		
	Helpful	474 (27.2%)		
	Very helpful	207 (11.8%)		
	Missing	344 (19.7%)		

Question	COVID-19 RELATED CHANGES AND RESTRICTIONS (PAST TWO WEEKS)	PARENT N=1741	YOUNG PERSON N=1708	r (p-value)*
Q59	**How much has the COVID-19 outbreak, and the resulting changes to daily life, led to positive changes in your family and community?**			
	Not much	363 (20.8%)		
	Slightly	326 (18.7%)		
	Moderately	364 (20.9%)		
	Quite a bit	275 (15.8%)		
	Very much	86 (4.9%)		
	Missing	327 (18.7%)		
Q60	**Why? (check all that apply)**			
	More quality time with family	544 (31.2%)		
	Less running around	407 (23.2%)		
	People looking out for each other	335 (19.2%)		
	Greater community outreach	186 (10.6%)		
	Environment less polluted	310 (17.8%)		
	Other	75 (4.3%)		
	Missing	1018 (58.4%)		
Q64 P/ Q29 YP	**How easy is it for you and your child to comply with the 'social distancing' restrictions put in place in your community? how easy is it for you to comply with the 'social distancing' restrictions put in place in your community?**			0.423 (<0.001)
	Very easy	406 (23.3%)	333 (19.5%)	
	Fairly easy	623 (35.7%)	432 (25.2%)	
	Somewhat difficult	282 (16.2%)	286 (16.7%)	
	Very difficult	94 (5.4%)	127 (7.4%)	
	Missing	336 (19.3%)	530 (31%)	
Q65	**How often are you getting outside your house for approved activities such as exercising, shopping for food, attending a medical appointment, work or school where necessary?**			
	Not at all	63 (3.6%)		
	Once a week	282 (16.2%)		
	Several times a week	526 (30.2%)		
	Once a day	365 (20.9%)		
	Several times a day	171 (9.8%)		
	Missing	334 (19.1%)		
Q46 P/ Q30 YP	**How often is your child getting outside your house for approved activities such as exercising, shopping for food, attending a medical appointment, work or school where necessary? how often are you getting outside your house for activities such as exercising, shopping for food, attending a medical appointment, work or school where necessary?**			0.655 (<0.001)
	Less than once a week	491 (28.2%)	517 (30.2%)	
	Several times a week	512 (29.4%)	370 (21.6%)	
	Once a day	330 (18.9%)	210 (12.3%)	
	Several times a day	105 (6%)	81 (4.7%)	
	Missing	303 (17.4%)	530 (31%)	

Question	EMPLOYMENT, FINANCES AND HOUSING (PAST TWO WEEKS)	PARENT N=1741	YOUNG PERSON N=1708	r (p-value)*
Q66	**Which one of the following best describes your current employment situation?**			
	Furloughed due to COVID-19	148 (8.5%)		
	Dismissed due to COVID-19	28 (1.6%)		
	Working more hours than usual	108 (6.2%)		
	Working fewer hours than usual	126 (7.2%)		
	Working usual hours (full-time or part-time) at normal place of work	181 (10.4%)		
	Working usual hours (full-time or part-time) from home	190 (10.9%)		
	Was not working outside the home	310 (17.8%)		
	Other	306 (17.5%)		
	Missing	344 (19.7%)		
Q67	**Which one of the following best describes the current employment situation of your partner? B missing**			
	Furloughed due to COVID-19	7 (0.4%)		
	Dismissed due to COVID-19	0 (0%)		
	Working more hours than usual	4 (0.2%)		
	Working fewer hours than usual	13 (0.7%)		
	Working usual hours (full-time or part-time) at normal place of work	27 (1.5%)		
	Working usual hours (full-time or part-time) from home	69 (3.9%)		
	Receiving Job Keeper allowance	2 (0.1%)		
	Was not working outside the home	7 (0.4%)		
	Other	9 (0.5%)		
	NA/missing	1603 (92%)		
Q68	**which one of the following best describes your financial situation at this point in the crisis?**			
	Living comfortably	238 (13.6%)		
	Doing alright	538 (30.9%)		
	Just getting by	427 (24.5%)		
	Finding it quite difficult	152 (8.7%)		
	Finding it very difficult	48 (2.7%)		
	Missing	338 (19.4%)		
Q69	**to what degree are you concerned about the stability of your housing situation?**			
	Not at all	704 (40.4%)		
	Slightly	296 (17%)		
	Moderately	179 (10.2%)		
	Very	111 (6.3%)		
	Extremely	108 (6.2%)		
	Missing	346 (19.7%)		
