# Supplementary material for: Emotional and behavioral outcomes among youths with mental disorders during the first Covid lockdown and school closures in England: a large clinical population study using health care record integrated surveys
Source: Soc Psychiatry Psychiatr Epidemiol. 2023 Jun 23;59(1):175–86. doi: 10.1007/s00127-023-02517-w (PMC10799796; doi:10.1007/s00127-023-02517-w)
Supplement: Supplementary file 2 — Supplementary file2 (PDF 1332 KB) [file 127_2023_2517_MOESM2_ESM.pdf]

SUPPLEMENTARY MATERIAL

Emotional and behavioral outcomes among youths with mental disorders during the first Covid lockdown and school closures in England: a large clinical population study using health care record integrated surveys. *European Child and Adolescent Psychiatry.*

Parlatini V. [1], Frangou L. [1]*, Zhang S. [1]*, Epstein S. [1], Morris A. [1], Grant C. [1,2], Zalewski L. [3,4], Jewell A. [4], Velupillai S. [3], Simonoff E.[1,4]¶, Downs J. [1,4]¶

* ¶ These authors equally contributed to the study

[1] Department of Child and Adolescent Psychiatry, Institute of Psychiatry, Psychology and Neuroscience, Kings College London, London, UK

[2] Department of Epidemiology & Public Health, University College London, London, UK

[3] Department of Biostatistics & Health Informatics, Institute of Psychiatry, Psychology and Neuroscience, King's College London, London, UK

[4] National Institute for Health Research (NIHR) Biomedical Research Centre, South London and Maudsley NHS Foundation Trust, London, UK

Corresponding author: valeria.parlatini@kcl.ac.uk

TABLE OF CONTENT

1. **SUPPLEMENTARY METHODS**

1.1 Timeframe	Page 3
Fig. S1 Study timeframe	Page 3
1.2 Data flow	Page 3
Fig. S2 Data flow diagram	Page 4
1.3 Socio-demographic characteristics	page 4
1.4 Diagnoses	Page 4
1.5 Factor analyses and composite scores	Page 5
1.6 Sensitivity analyses	Page 5
1.7 Analyses on education	Page 5

2. **SUPPLEMENTARY RESULTS**

2.1 Flow chart and sample characteristics by diagnosis	Page 6
Fig. S4 Survey data flow and Venn diagram	Page 6
2.2 Factor analyses and composite scores	Page 6
Fig. S3 Scree plots and factor loadings	Page 7
2.3 Sensitivity analyses	Page 8
2.4 Analyses on education	Page 8

3. **SUPPLEMENTARY TABLES**

Table S1 Correlation analyses	Page 9
Table S2 Baseline characteristics in the three main diagnostic groups	Page 10
Tables S3-4 Unadjusted regressions	Page 11
Tables S5-8 Adjusted regressions	Page 13
Table S9 Sensitivity analyses	Page 17
Table S10 Education and mental health	Page 17

4. **LIST OF ABBREVIATIONS** Page 18

5. **SUPPLEMENTARY REFERENCES** Page 18

1. SUPPLEMENTARY METHODS

1.1 Timeframe

The survey was first rolled out between the 15thJune 2020 and 2nd September 2020. During this time, measures of social distancing were easing after a period of national lockdown (Fig. S1). From June there was a gradual re-opening of schools, initially to reception (age 4-5), year 1 (age 5-6) and year 6 (age 10-11) followed two2 and weeks later by years 10 (age 14-15) and 12 (age 16-17). However, school attendance was not compulsory during this time and, nationally, the peak school attendance was only 17.5% (https://www.childrenscommissioner.gov.uk/wp-content/uploads/2020/12/cco-briefing-on-school-attendance-since-september). Education was suspended for the summer holiday from the 23rdJuly, and most children and young people (CYP) returned to in-person education at the start of the new school year on the 1stSeptember.

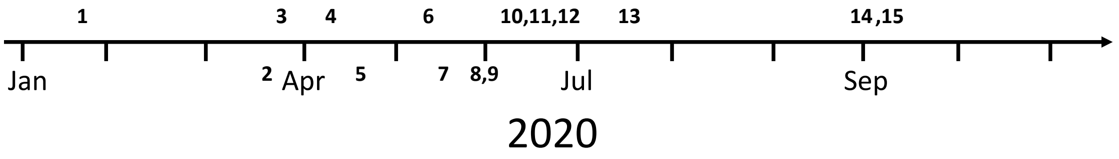

#Event

1. First known suspected case in UK
2. Schoolchildren required to stay home from school
3. UK lockdown begins - school, non-essential shops and places of worship shut. Outdoor exercise limited to once per day. No contact with people outside the household
4. Furlough scheme starts
5. Furlough scheme extended till 07/2020
6. Unlimited outdoor exercise
7. Furlough scheme extended till 11/2020
8. Groups of 6 allowed to meet outdoors

#Event

9. Year 1 and 6 return to schools
10. Non-essential shops reopen
11. Places of worship reopen
12. Year 10 and 12 return to schools
13. Schooling stops for summer
14. Schools reopen
15. Data collection completed

Fig S1. Study timeframe. The survey was first rolled out between the 15th June 2020 and 2nd September 2020. During this time, measures of social distancing were easing after a period of national lockdown and schools gradually re-opened before closing again for the summer holidays (23rd July to 31st August). Most CYP returned to in-person education from the 1st September.

1.2 Data flow

Caregivers (as identified from electronic health records, EHRs) and young people above 16 were contacted directly and received a unique link to the survey via text or email through GOV.UK Notify, a service provided by the UK Government Digital Service. Caregivers were asked to forward the survey to their child (below 16) if thought appropriate. Follow-up notifications were sent after three days to remind those that had not opted out to complete the survey. Individual responses were temporarily hosted on the Qualtrics platform before being fed back into EHRs. Clinical and socio-demographic data were extracted from CYP's EHRs through the Clinical Record Interactive Search (CRIS) tool and integrated with survey responses for statistical analysis (Fig. S2).

The study was commissioned by our Trust and there was clinical and managerial agreement in place to roll out the survey under Regulation 3(2) and Regulation 3(3) of the Health Service Control of Patient Information Regulations 2002 (COPI). These permit the processing and dissemination of confidential patient information for the purposes of research, protecting public health, providing healthcare services to the public and monitoring and managing the Covid outbreak and incidents of exposure. Extraction and analysis of deidentified outcome data were carried out using the CRIS platform and security model approved by Oxford Research Ethics Committee C (reference 18/SC/0372).

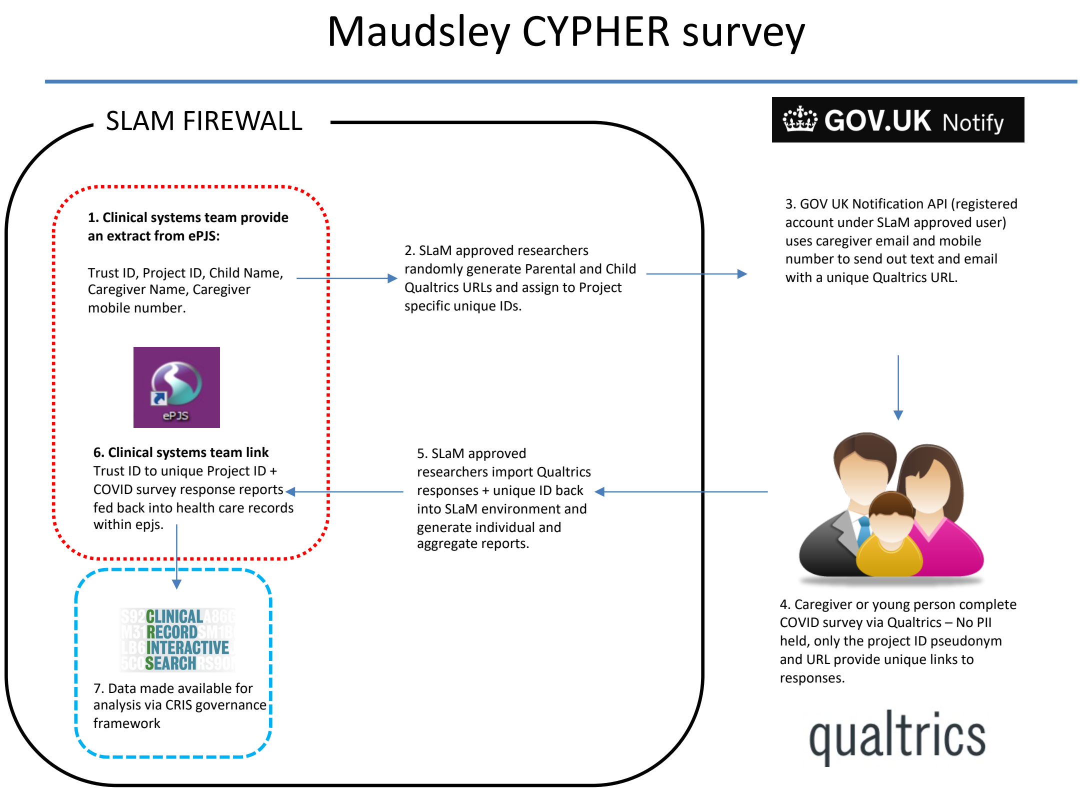

Fig. S2 Data flow diagram. Caregivers and young people were contacted via text or email via GOV.UK Notify with a unique link to the survey. Individual responses were temporarily hosted on the Qualtrics platform before being fed back into clinical records. Clinical and socio-demographic data were extracted from CYP's health records through the Clinical Record Interactive Search (CRIS) tool and integrated with survey responses for statistical analysis.

1.3 Socio-demographic characteristics

Socio-demographic characteristics included age at the first lockdown (23rd March 2020), sex, ethnicity, and neighborhood deprivation. These characteristics were selected because they have been associated with mental health outcomes in previous studies [1]. Ethnicity was defined according to the guidelines of the UK Office for National Statistics and collapsed into 5 categories: White, Black, Asian, Mixed, Other, Non-stated. Neighborhood deprivation is a proxy for socio-economic deprivation and was derived from Index of Multi-deprivation (IMD) scores, which are provided by the Office of National Statistics (ONS) at low level small area output as a composite of 7 domains, including income, employment, health and disability, education, skills and training, barriers to housing and services, living environment, and crime (https://assets.publishing.service.gov.uk/government/uploads/system/uploads/attachment_data/file/465791/English_Indices_of_Deprivation_2015_-_Statistical_Release.pdf). For ease of interpretation, we converted the IMD scores into four categories of increasing socio-economic deprivation [2].

1.4 Diagnoses

The most represented diagnoses in our CAMHS population were: Attention Deficit Hyperactivity Disorder-ADHD (i.e. hyperkinetic disorders, ICD-10 codes F90.0, F90.1, F90.2, F90.8, F90.9); Autism Spectrum Disorder-ASD (F84.0, F84.1, F84.5, F84.9); and emotional disorders-EmD, which include depressive disorders, anxiety disorders, Post-Traumatic Stress Disorder-PTSD, and Obsessive-Compulsive Disorder-OCD (F32, F33, F34, F38, F40-49, F93, F94). For group comparisons, as co-occurrent disorders are frequent, diagnoses were organized according to a hierarchy according to the main treatment pathway: CYP were assigned to the ASD group regardless of whether they also had ADHD and/or EmD, whilst CYP were assigned to the ADHD group regardless of whether they had secondary emotional disorders. In the regression analyses we considered the independent effect of any of the three main diagnoses (without hierarchy), but also included additional diagnoses, if present, as covariates in the adjusted model for the three diagnostic groups, as they may contribute

to variation in the effect of the main exposure. Co-occurring disorders included ADHD, ASD, and EmD (as defined above) and intellectual disability (ID, F70-79).

1.5 Factor analyses and composite scores

We used factor analysis to test whether the structure underlying parent responses on child's symptoms (12 questions) reflected a smaller set of clinical domains and separated emotional from behavioral symptoms, as originally intended when survey questions were developed. Questions on child's symptoms included anxiety, general worries, sadness, anhedonia, fatigue, loneliness, inattention, restlessness, irritability, aggression, worries about being infected and worries about significant others being infected. We did not consider quality of sleep, as this was not rated on a 5-point Likert scale reflecting increasing levels of difficulties as the remaining questions. The same procedure was followed to test the structure underlying the 17 questions on parental stress. These included parent's emotional and behavioral symptoms (anxiety, general worries, sadness, anhedonia, fatigue, loneliness, inattention, restlessness, irritability, worries about being/or others being infected); work-family balance; family's ability to cope; support from family members; support from social network; quality of relationship with partner; concerns about finances; and concerns about housing. Finally, additional composite scores were calculated for the remaining survey responses on contextual factors, where these could be aggregated according to common themes. See section 2.2. for results.

1.6 Sensitivity analyses

We carried out two sensitivity analyses. The first verified whether the association between parental and child's mental health was due to a shared-method variance effect, by testing whether the association held when we replaced parent-rated outcomes with child-rated ones in the fully adjusted regressions. The second tested whether differences in mental health outcomes among the three main diagnostic groups held independently from contextual and socio-demographic factors, by using Wald tests to contrast the three main diagnostic groups as predictors of mental health outcomes in unadjusted and fully adjusted regression models.

1.7 Analyses on education

We first analyzed clinico-demographic characteristics according to education modality (in-person vs remote) in the whole sample through descriptive statistics. To this purpose, we collapsed the four modalities of school attendance (in-person full time, in-person part-time, remote part-time, remote full time) into two categories (in person and remote). We then tested whether the three main diagnostic groups differed in education modality (through chi-squared test) and in remote education enjoyment/engagement (through one-way ANOVAs and post-hoc t-tests were appropriate). We also tested whether education enjoyment differed according to education modality in the whole sample and in the three groups (through t-tests). Finally, we investigated whether education enjoyment/engagement were associated with emotional or behavioral change (through unadjusted regressions).

2. SUPPLEMENTARY RESULTS

2.1 Flow chart and sample characteristics by diagnosis.

Between June and September 2020, 2503 caregivers and/or young people (46.5% of the sampling frame) responded to the Maudsley CYPHER survey for a total of 1741 parent responses and 1708 CYP responses. As shown in the Venn diagram, ADHD was the most common single diagnosis (N=568), followed by EmD (N=336) and ASD (N=318). The co-occurrence of ADHD and ASD was the most common (N=376) (Fig. S4). Please see Table S1 for socio-demographic characteristics by diagnostic group.

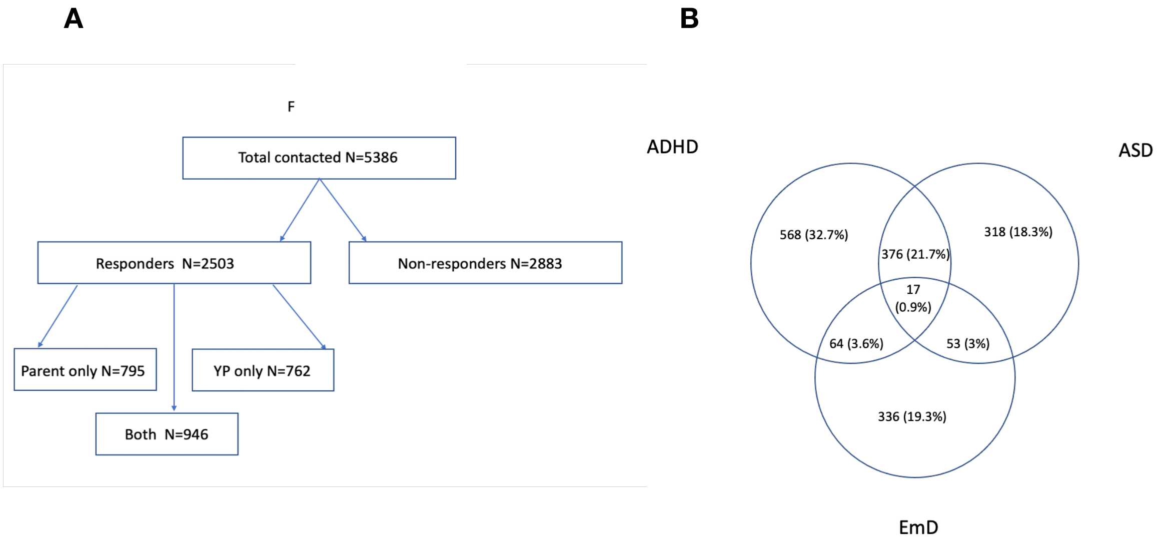

Fig. S4 Flow chart and Venn diagram. Between June and September 2020, 1741 parent responses and 1708 CYP responded to the Maudsley CYPHER survey. As shown in the Venn diagram, ADHD was the most common single diagnosis, followed by EmD and ASD. The co-occurrence of ADHD and ASD was the most common.

2.2 Factor analyses and composite scores

The first factor analysis tested the structure underlying parent responses on child's symptoms (12 questions). The preliminary factor analysis with no rotation identified three factors with eigenvalue above 1, which explained 64.5% of the variance of the reported clinical symptoms. Scree plot confirmed the three-factor solution; thus, a second factor analysis was performed, this time with a fixed number of three factors to extract and with an oblique promax rotation, as recommended for correlated variables in large datasets. Three groups of symptoms were identified: emotional symptoms (anhedonia, sadness, general worries, anxiety, fatigue, and loneliness); behavioral symptoms (restlessness, inattention, irritability and aggression); and Covid-related worries (worries about being infected and about significant others being infected) (Fig. S3).

Scores from individual survey responses within the first two factors were summed to calculate the corresponding composite outcome measures: total emotional score and total behavioral score. Higher scores reflected increased severity.

The same procedure was followed to test the structure underlying the 17 questions on parental stress. The first unrotated factor analysis and scree plot identified three factors with eigenvalues above 1, explaining 49% of the variance in parental stress. The second factor analysis was performed with a fixed number of three factors and an oblique promax rotation (Fig. S3).

Scores from individual survey responses within the three factors were summed to calculate the corresponding composite scores: poor parental mental health (including 10 questions on emotional and behavioral symptoms); lack of family support (family's ability to cope; support from family members; support from social network); and parental concerns (worries about finances and housing). Responses on work-family balance and quality of relationship with partner did not load on any specific factor and were not included. Higher scores reflected increased difficulties.

Additional composite scores were calculated for the remaining survey responses on contextual factors, where these could be aggregated according to common themes. These included: housing inadequacy (indoor and outdoor space); challenges with education (as reflected by poor education engagement and enjoyment); perceived inadequacy of child's mental health care (quality, quantity, and needs met) and quality of child's relationships (with parents, siblings and friends). Covid exposure and physical health questions were non considered due to the limited number of CYP having a suspected infection (< 4%). As the above themes were surveyed by less than four questions each, we did not perform factor analyses but calculated composite scores when individual responses were correlated. Results of the correlation analyses are reported in Table S1.

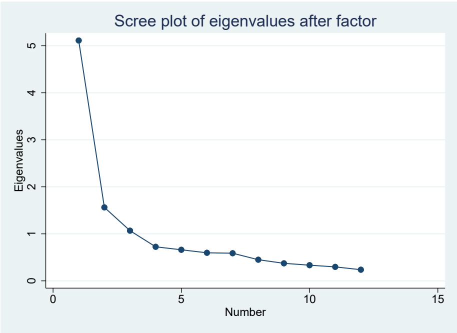

FACTOR ANALYSIS 1

VARIABLE	FACTOR 1 - EMOTIONAL SYMPTOMS	FACTOR 2 - BEHAVIOURAL SYMPTOMS	FACTOR 3 - COVID-RELATED WORRIES
Anhedonia	0.745		
Sadness	0.762		
General worries	0.422		
Anxiety	0.519		
Fatigue	0.772		
Loneliness	0.75		
Restlessness		0.714	
Inattention		0.676	
Irritability		0.758	
Aggression		0.926	
Worried about being infected			0.944
Worried about others being infected			0.932

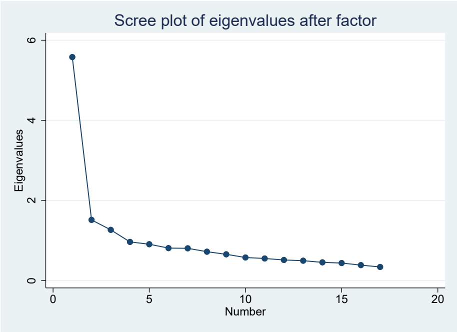

FACTOR ANALYSIS 2

VARIABLE	FACTOR 1 - EMOTIONAL SYMPTOMS	FACTOR 2 - BEHAVIORAL SYMPTOMS	FACTOR 3 - COVID-RELATED WORRIES
Anhedonia	0.745		
Sadness	0.762		
General worries	0.422		
Anxiety	0.519		
Fatigue	0.772		
Loneliness	0.75		
Restlessness		0.714	
Inattention		0.676	
Irritability		0.758	
Aggression		0.926	
Worried about being infected			0.944
Worried about others being infected			0.932

Fig. S3 Scree plots and factor loadings. The first factor analysis included parent responses on child's symptoms (12 questions) and identified three factors: emotional symptoms (anhedonia, sadness, general worries, anxiety, fatigue, and loneliness); behavioral symptoms (restlessness, inattention, irritability and aggression); and Covid-related worries (worries about being infected and about significant others being infected). The second factor analysis included 17 questions on parental stress, i.e. mental health symptoms; work-family balance; family's ability to cope; support from family members; support from social network; quality of relationship with partner; concerns about finances; and concerns about housing. Three factors were identified: parental mental health (including 10 questions on emotional and behavioral symptoms); lack of family support (family's ability to cope; support from family members; support from social network); and parental concerns (worries about finances and housing). Responses on work-family balance and quality of relationship with partner did not load on any specific factor and were not included.

2.3 Sensitivity analyses

The first sensitivity analysis aimed at testing whether the association between parents' and CYP's mental health outcomes was due to a shared-method variance effect. The observed association held when we replaced parent-rated outcomes with young person-rated ones in the fully adjusted regressions for total emotional score (coeff=0.092, p=0.002), total behavioral score (coeff=0.054, p=0.015) and behavioral change (coeff=0.014, p=0.024); but lost significance for emotional change (coeff=0.012, p=0.082). This suggests that, overall, the observed associations were unlikely due to shared-method variance; however, a parent's view on their child's emotional change may be more influenced by their own mental state than that on other outcomes.

In the second sensitivity analysis, when we contrasted the three main diagnostic groups as predictor of each mental health outcome in unadjusted and fully adjusted regression models, most of the observed differences between diagnostic groups held independently from contextual and socio-demographic factors (Table S9). In brief, considering total emotional difficulties, there was a significant difference between ADHD and ASD (p<0.001 and p=0.001 respectively) in the unadjusted regression model. The former association retained significance in the fully adjusted model (p=0.006). Significant differences among the three groups were observed for emotional change in the unadjusted model (respectively ADHD vs ASD p=0.003; ASD vs EmD p<0.001 and ADHD vs EmD p=0.009). The first two differences remained significant in the fully adjusted model (p=0.016 and p<0.001 respectively). Considering total behavioral difficulties, significant differences were observed between ADHD and EmD (both p<0.001) in the unadjusted regression model, which persisted in the fully adjusted model (p=0.001 and <0.001 respectively). Finally, significant differences in behavioral change were observed between ADHD and EmD and between ASD and EmD (both p<0.001) in the unadjusted model. The latter retained significance in the fully adjusted model (p=0.007). Taken together these findings suggest that most of the observed differences among diagnostic groups were independent from contextual and socio-demographic factors.

2.4 Analyses on education

Considering clinico-demographic characteristics according to education modality (in-person vs remote), in-person education was more prevalent among primary school children (51% vs 32% and 26% in secondary and college students respectively) and in boys (46% vs 38%). We did not observe significant differences in education modality among diagnostic groups, but ANOVAs showed a significant effect of diagnosis on remote education engagement and enjoyment (F(2,521)=5.62, p=0.003 and 3.61, p=0.027). Post-hoc t-tests revealed that CYP with ASD had lower educational enjoyment than those with EmD (t(322)=2.664, p=0.008), and both those with ASD and ADHD had lower educational engagement than those with EmD (t(321)=3.263, p=0.001 and t(300)=2.058, p=0.040). Further, CYP with ASD and ADHD enjoyed remote education less than in-person (t(355)=-2.38, p<0.05 and t(425)=-2.83, p<0.01 respectively). Finally, we observed an inverse association between education engagement/enjoyment and emotional and behavioral change in the whole sample and in the three main diagnostic groups (apart from emotional change for those with EmD) (Table S10).

3. SUPPLEMENTARY TABLES

Table S1. Correlation analyses. This table shows correlation results for survey responses on contextual variables aggregated according to four common themes: quality of child's relationships (with parents, siblings and friends); housing inadequacy (indoor and outdoor space); educational challenges (as reflected by poor education engagement and enjoyment); perceived inadequacy of child's mental health care (quality, quantity, and needs met). As the above themes were surveyed by less than four questions each, we did not perform factor analyses but calculated composite scores when individual responses were correlated.

COMPOSITE SCORES	CORRELATIONS
**Poor child's relationships**	
1) Relationships with parents	1-2: r=0.640***
2) Relationships with siblings	2-3: r=0.323***
3) Relationships with friends	1-3: r=0.351***
**Housing inadequacy**	
1) Limited space indoor	1-2: r=0.152***
2) Limted space outdoor	
**Educational challenges**	
1) Education enjoyment	1-2: r=582***
2) Education angagement	
**Perceived inadequacy of MH care**	
1) Quality	1-2: r=0.415***
2) Quantity	2-3: r=0.339***
3) Needs met	1-3: r=0.516***

Table S2. Baseline characteristics in the three main diagnostic groups. This table shows pre-pandemic socio-demographic characteristics (including age, sex, ethnicity, and neighborhood deprivation) for each of the main diagnostic groups (responders only).

	**ADHD** **N=632**	**ASD** **N=764**	**EmD** (a) **N=336**
**Age at lockdown** Mean (SD)	12.8 (3.1)	12.9 (3.1)	15 (2.6)
**School age groups** N(%)			
Primary school	239 (37.8%)	292 (38.2%)	44 (13.1%)
Secondary school	244 (38.6%)	275 (35.9%)	122 (36.3%)
College	149 (23.5%)	197 (25.7%)	170 (50.6%)
**Sex** N(%) (b)			
Male	451 (71.4%)	509 (66.8%)	87 (26%)
Female	180 (28.5%)	253 (33.2%)	247 (73.9%)
**Ethnicity** N(%)			
White	295 (46.6%)	391 (51.1%)	186 (55.3%)
Black	124 (19.6%)	137 (17.9%)	58 (17.2%)
Asian	6 (0.9%)	14 (1.8%)	14 (4.1%)
Mixed	72 (11.3%)	79 (10.3%)	29 (8.6%)
Other	11 (1.7%)	12 (1.5%)	4 (1.1%)
Non-stated	124 (19.6%)	131 (17.1%)	45 (13.3%)
**Neighborhood deprivation** N(%) (b)			
Least deprived	113 (18.4%)	184 (24.6%)	74 (22.8%)
2nd least deprived	111 (18.1%)	147 (19.7%)	70 (21.6%)
3rd least deprived	126 (20.5%)	122 (16.3%)	69 (21.3%)
2nd most deprived	141 (23%)	119 (15.9%)	61 (18.8%)
Most deprived	122 (19.9%)	174 (23.3%)	50 (15.4%)

(a)= Emotional disorders include depressive disorders, anxiety disorders, PTSD, and OCD
(b)= The following variables have missing data: sex (missing N=255), neighborhood deprivation (missing N=49).

Table S3 Unadjusted regressions for emotional outcomes. This table shows the results of the unadjusted linear regression analyses, which identified pre-Covid socio-demographic characteristics, Covid-related contextual factors and co-occurrent disorders associated with total emotional score and emotional change in the whole sample with parent responses and in the three main diagnostic groups. Numbers of observations are reported in footnotes.

	WHOLE SAMPLE	ADHD	ASD	EmD	WHOLE SAMPLE	ADHD	ASD	EmD
	Unadjusted analysis Coeff (p-value)	Unadjusted analysis Coeff (p-value)	Unadjusted analysis Coeff (p-value)	Unadjusted analysis Coeff (p-value)	Unadjusted analysis Coeff (p-value)	Unadjusted analysis Coeff (p-value)	Unadjusted analysis Coeff (p-value)	Unadjusted analysis Coeff (p-value)
	TOTAL EMOTIONAL SCORE				EMOTIONAL CHANGE			
**Age at lockdown**	.261***	0.211***	.184*	.375**	-0.01	-0.004	-0.02	0.013
**Sex**	.648*	0.461	0.891	0.662	-.134*	-0.046	-0.019	-0.106
**Ethnicity**								
White	Reference	Reference	Reference	Reference	Reference	Reference	Reference	Reference
Black	-1.298 ***	-1.272*	-1.25	-2.169*	-0.078	-0.168	0.215	-0.301
Asian	1 (.371)	1.611	-0.067	0.881	-0.073	0.054	-0.213	0.323
Mixed	-.995*	-0.52	-1.028	-1.464	-0.071	-0.092	-0.079	-0.079
Other	-1.284	-0.797	1.332	2.631	-.454*	-0.422	-0.313	-0.176
Non-stated	-1.118**	-0.455	-1.474*	-1.057	-0.085	-0.116	-0.022	-0.365
**Neighbourhood deprivation**								
Least deprived	Reference	Reference	Reference	Reference	Reference	Reference	Reference	Reference
2nd least deprived	-0.228	-0.857	-0.133	1.02	-0.006	-0.173	0.083	0.066
3rd least deprived	-0.606	-0.549	0.798	0.377	-0.019	-0.021	0.215	0.132
2nd most deprived	0.084	-0.237	0.379	0.765	0.134	0.094	0.22	0.345
Most deprived	-0.569	-0.717	-0.534	0.271	0.031	-0.03	0.132	0.214
**Contextual factors**								
Housing inadequacy	.651***	0.615***	0.631**	0.721**	.155***	0.124***	0.159***	0.181**
Poor parental MH	.321***	0.31***	0.334***	0.304***	.050***	0.042***	0.053***	0.05***
Lack of family support	.613***	0.522***	0.557***	0.542***	.110***	0.072***	0.103***	0.1**
Parental concerns	.547***	0.556***	0.541***	0.601***	.117***	0.081***	0.145***	0.131***
Challenges with education	.739***	0.696***	0.816***	0.6***	.121***	0.115***	0.115***	0.047
Perceived inadequacy of child's MH care	.348***	0.414***	0.219	0.512*	.034***	0.053**	0.012	0.07
Limited outdoor time	1.3***	1.234***	1.337***	1.243***	.163***	0.171***	0.141**	0.232**
Difficulty with 'social distancing'	.88***	0.787**	0.69*	1.07*	.236***	0.183***	0.231***	0.286**
**Comorbidities**								
ADHD			-0.403	0.464			0.139	0.171
ASD		1.293 **		0.263		0.267**		0.093
EmD		1.603*	0.329			-0.168	-.35*	
ID		-2.188*	-1.326	-4.089		-0.089	-0.051	-0.141

Number of observations:

Whole sample: total emotional score, range 1385-1551; emotional change, range 1387-1153 (lowest for lack of family support, highest for ethnicity).
ADHD: total emotional score, range 576-654; emotional change, range 578-656 (lowest for parental concerns, highest for age, ethnicity and comorbidities).
ASD: total emotional score, range 432-482; emotional change, range 434-484 (lowest for parental concerns, highest for age, sex, ethnicity and comorbidities).
EmD: total emotional score, range 253-279; emotional change, range 254-280 (lowest for parental concerns, highest for age, ethnicity and comorbidities).

Table S4 Unadjusted regressions for behavioral outcomes. This table shows the results of the unadjusted linear regression analyses, which identified pre-Covid socio-demographic characteristics, Covid-related contextual factors and co-occurrent disorders associated with total behavioral score and behavioral change in the whole sample with parent responses and in the three main diagnostic groups. Numbers of observations are reported in footnotes.

	WHOLE SAMPLE	ADHD	ASD	EmD	WHOLE SAMPLE	ADHD	ASD	EmD
	Unadjusted analysis Coeff (p-value)	Unadjusted analysis Coeff (p-value)	Unadjusted analysis Coeff (p-value)	Unadjusted analysis Coeff (p-value)	Unadjusted analysis Coeff (p-value)	Unadjusted analysis Coeff (p-value)	Unadjusted analysis Coeff (p-value)	Unadjusted analysis Coeff (p-value)
	TOTAL BEHAVIOURAL SCORE				BEHAVIOURAL CHANGE			
**Age at lockdown**	-.278 ***	-0.227***	-0.304***	-0.197*	-.035***	-0.023*	-0.043**	-0.0004
**Sex**	-1.219 ***	-0.437	-0.363	-0.151	-.202***	-0.077	-0.018	-0.151
**Ethnicity**								
White	Reference	Reference	Reference	Reference	Reference	Reference	Reference	Reference
Black	-.571*	-0.494	-0.97	-0.324	0.007	-0.119	-0.012	0.03
Asian	-0.362	-1.093	-0.223	1.25	-0.269	-0.289	-0.439	0.105
Mixed	-0.3	0.318	-1.435*	-1.124	-0.011	0.063	-0.061	0.144
Other	-1.6	-1.729	-1.223	0.875	-0.412	-0.425	0.426	0.355
Non-stated	-0.06	-0.219	-0.186	-0.518	-0.043	-0.096	-0.007	-0.363
**Neighbourhood deprivation**								
Least deprived	Reference	Reference	Reference	Reference	Reference	Reference	Reference	Reference
2nd least deprived	-0.102	-1.119*	-0.259	0.697	0.015	-0.212	0.091	0.076
3rd least deprived	0.176	0.163	0.933	0.851	-0.01	-0.009	0.196	0.099
2nd most deprived	.779*	-0.034	0.342	1.628*	0.142	0.052	0.202	0.272
Most deprived	0.064	-0.409	-0.59	0.642	0.047	-0.012	0.067	0.132
**Contextual factors**								
Housing inadequacy	.817***	0.648***	0.728***	0.655**	.166***	0.162***	0.164***	.119*
Poor parental MH	.242***	0.230***	0.232***	.197***	.050***	0.043***	0.05***	0.046***
Lack of family support	.576***	0.410***	0.512***	0.349***	.120***	0.09***	0.132***	0.1***
Parental concerns	.453***	0.404***	0.486***	0.401***	.111***	0.095***	0.12***	0.102**
Challenges with education	.716***	0.534***	0.605***	0.608***	.131***	.106***	0.133***	.078**
Perceived inadequacy of child's MH care	.312***	0.404***	0.23*	0.394**	0.027	0.055**	0.008	0.016
Limited outdoor time	.151***	0.212	0.195	0.138	.092***	0.102*	0.144**	0.128
Difficulty with 'social distancing'	1.276***	1.292***	1.079***	0.756*	.256***	0.207***	.214***	.255**
**Comorbidities**								
ADHD			1.418***	1.381**			0.167	0.262
ASD		0.985**		-0.417		0.154*		0.046
EmD		-1.002	-2.535***			-0.181	-.39*	
ID		0.038	-0.008	-0.57		-0.387	-0.09	0.023

Number of observations:
Whole sample: total behavioral score, range 1387-1553; behavioral change, range 1388-1156 (lowest for lack of family support, highest for ethnicity).
ADHD: total behavioral score, range 577-655; behavioral change, range 579-659 (lowest for parental concerns, highest for age, ethnicity and comorbidities).
ASD: total behavioral score range 433-483; behavioral change, range 434-484 (lowest for parental concerns, highest for age, sex, ethnicity and comorbidities).
EmD: total behavioral score, range 254-280; behavioral change, range 255-281 (lowest for parental concerns, highest for age, ethnicity and comorbidities).

Table S5. Adjusted regressions for total emotional score. This table shows the results of the linear regression analyses progressively adjusting for pre-Covid socio-demographic characteristics and Covid-related contextual factors. In the fully adjusted models for the three main diagnostic groups, we also included co-occurrent disorders, if present, as covariates. Numbers of observations are reported in footnotes.

	TOTAL EMOTIONAL SCORE															
	WHOLE SAMPLE								ADHD							
	Demographic adjustment				Demographic and contextual adjustment				Demographic adjustment				Demographic and contextual adjustment			
	Coeff	95%CI		p	Coeff	95%CI		p	Coeff	95%CI		p	Coeff	95%CI		p
**Age at lockdown**	0.229	0.147	0.311	***	0.190	0.114	0.267	***	0.203	0.079	0.327	**	0.188	0.066	0.310	**
**Sex**	0.304	-0.227	0.835		0.733	0.255	1.211	**	0.251	-0.626	1.127		0.091	-0.702	0.883	
**Ethnicity**																
White	Reference				Reference				Reference				Reference			
Black	-1.214	-1.947	-0.482	**	-1.043	-1.721	-0.365	**	-1.310	-2.416	-0.204	*	-1.190	-2.211	-0.168	*
Asian	0.684	-1.503	2.871		-0.551	-2.373	1.271		0.648	-4.293	5.590		-0.712	-4.883	3.460	
Mixed	-0.816	-1.679	0.047		-0.816	-1.587	-0.045	*	-0.349	-1.678	0.980		-0.635	-1.851	0.581	
Other	-1.585	-3.774	0.604		-1.101	-3.123	0.920		-1.430	-4.428	1.568		-0.108	-2.701	2.486	
Non-stated	-0.800	-1.526	-0.074	*	-1.168	-1.824	-0.513	***	-0.204	-1.242	0.835		-0.666	-1.611	0.279	
**Neighbourhood deprivation**																
Least deprived	Reference				Reference				Reference				Reference			
2nd least deprived	-0.061	-0.819	0.697		-0.224	-0.893	0.444		-0.768	-1.959	0.422		-0.306	-1.375	0.763	
3rd least deprived	-0.236	-1.046	0.573		-0.217	-0.954	0.520		-0.269	-1.516	0.978		0.275	-0.863	1.414	
2nd most deprived	0.326	-0.492	1.145		-0.463	-1.206	0.281		-0.046	-1.303	1.210		-0.378	-1.521	0.764	
Most deprived	-0.205	-0.996	0.586		-0.643	-1.362	0.077		-0.456	-1.630	0.717		-0.361	-1.439	0.717	
**Contextual factors**																
Housing inadequacy					0.033	-0.192	0.259						-0.062	-0.378	0.253	
Poor parental MH					0.254	0.217	0.290	***					0.249	0.193	0.305	***
Lack of family support					0.128	0.016	0.240	*					0.088	-0.084	0.259	
Parental concerns					0.052	-0.090	0.194						0.122	-0.085	0.330	
Challenges with education					0.406	0.298	0.513	***					0.350	0.184	0.515	***
Perceived inadequacy of child's MH care					0.151	0.027	0.276	*					0.215	0.027	0.402	*
Limited outdoor time					0.760	0.501	1.018	***					0.681	0.301	1.062	***
Difficulty with 'social distancing'					0.362	0.082	0.642	*					0.325	-0.105	0.756	
**Comorbidities**																
ADHD																
ASD													0.947	0.194	1.700	*
EmD													0.926	-0.368	2.220	
ID													-3.040	-5.014	-1.066	**

	TOTAL EMOTIONAL SCORE															
	ASD								EmD							
	Demographic adjustment				Demographic and contextual adjustment				Demographic adjustment				Demographic and contextual adjustment			
	Coeff	95%CI		p	Coeff	95%CI		p	Coeff	95%CI		p	Coeff	95%CI		p
**Age at lockdown**	0.139	-0.016	0.294		0.136	-0.019	0.291		0.278	0.040	0.517	*	0.187661	-0.04838	0.423703	
**Sex**	0.675	-0.320	1.669		0.595	-0.300	1.491		0.620	-0.665	1.905		0.849324	-0.44849	2.147138	
**Ethnicity**																
White	Reference				Reference				Reference				Reference			
Black	-1.255	-2.669	0.158		-0.754	-2.058	0.550		-1.981	-3.926	-0.036	*	-2.350225	-4.29323	-0.40722	*
Asian	-0.068	-4.259	4.124		0.853	-2.739	4.446		1.199	-2.425	4.822		0.027103	-3.16535	3.219559	
Mixed	-0.962	-2.551	0.628		-1.107	-2.568	0.355		-1.447	-3.630	0.737		-1.361029	-3.46411	0.74205	
Other	0.631	-3.989	5.252		0.734	-5.395	6.863		2.394	-2.753	7.540		0.527912	-4.75762	5.813448	
Non-stated	-1.203	-2.529	0.124		-1.123	-2.318	0.072		-1.000	-2.945	0.944		-1.496909	-3.3305	0.336683	
**Neighbourhood deprivation**																
Least deprived	Reference				Reference				Reference				Reference			
2nd least deprived	0.023	-1.391	1.438		-0.048	-1.312	1.216		1.038	-0.709	2.786		0.424067	-1.21257	2.060704	
3rd least deprived	0.948	-0.572	2.469		0.320	-1.088	1.727		0.866	-1.076	2.808		0.670825	-1.22814	2.569788	
2nd most deprived	0.590	-0.945	2.126		0.371	-1.031	1.773		1.101	-0.781	2.984		-0.042885	-1.86572	1.779953	
Most deprived	-0.137	-1.504	1.229		-1.204	-2.464	0.056		0.795	-1.326	2.916		0.261769	-1.76464	2.288173	
**Contextual factors**																
Housing inadequacy					-0.145	-0.553	0.264						0.321152	-0.29114	0.933441	
Poor parental MH					0.289	0.221	0.358	***					0.254305	0.161502	0.347108	***
Lack of family support					-0.002	-0.212	0.208						-0.104609	-0.40658	0.197364	
Parental concerns					0.161	-0.098	0.420						0.026176	-0.33115	0.383499	
Challenges with education					0.448	0.248	0.647	***					0.346946	0.091925	0.601968	**
Perceived inadequacy of child's MH care					0.075	-0.148	0.298						0.171163	-0.20309	0.54542	
Limited outdoor time					0.727	0.227	1.227	**					0.972917	0.268848	1.676987	**
Difficulty with 'social distancing'					0.282	-0.220	0.784						1.114956	0.284091	1.945821	**
**Comorbidities**																
ADHD					-0.422	-1.310	0.466						-0.060821	-1.58111	1.459466	
ASD													0.577463	-1.03393	2.188853	
EmD					0.573	-0.932	2.077									
ID					-2.141	-3.681	-0.600	***					-4.317634	-9.52839	0.893127	

Number of observations:

Whole sample: total emotional score, demographic adjustment N=1500, demographic and contextual adjustment N=1289

ADHD: total emotional score, demographic adjustment N=638, demographic and contextual adjustment N=539

ASD: total emotional score, demographic adjustment N=469, demographic and contextual adjustment N=405

EmD: total emotional score, demographic adjustment N=265 demographic and contextual adjustment N=228

Table S6. Adjusted regressions for emotional change. This table shows the results of the linear regression analyses progressively adjusting for pre-Covid socio-demographic characteristics and Covid-related contextual factors. In the fully adjusted models for the three main diagnostic groups, we also included co-occurrent disorders, if present, as covariates. Numbers of observations are reported in footnotes.

	EMOTIONAL CHANGE															
	WHOLE SAMPLE								ADHD							
	Demographic adjustment				Demographic and contextual adjustment				Demographic adjustment				Demographic and contextual adjustment			
	Coeff	95%CI		p	Coeff	95%CI		p	Coeff	95%CI		p	Coeff	95%CI		p
**Age at lockdown**	-0.007	-0.024	0.010		-0.014	-0.032	0.004		-0.00559	-0.02979	0.018601		-0.01516	-0.04126	0.010949	
**Sex**	-0.117	-0.227	-0.008	*	-0.009	-0.119	0.102		-0.02204	-0.19259	0.1485		0.046907	-0.12289	0.216706	
**Ethnicity**																
White	Reference				Reference				Reference				Reference			
Black	-0.107	-0.259	0.044		-0.077	-0.234	0.079		-0.19611	-0.41173	0.019503		-0.1588	-0.37775	0.060159	
Asian	-0.074	-0.527	0.379		-0.266	-0.686	0.155		0.049228	-0.91477	1.013227		0.156712	-0.73701	1.05044	
Mixed	-0.098	-0.278	0.081		-0.070	-0.249	0.109		-0.09034	-0.3494	0.168714		-0.12389	-0.38447	0.136693	
Other	-0.424	-0.878	0.029		-0.249	-0.716	0.218		-0.43462	-1.01948	0.150238		-0.22552	-0.78147	0.330426	
Non-stated	-0.110	-0.261	0.040		-0.195	-0.347	-0.044	*	-0.13522	-0.3381	0.067665		-0.22575	-0.42824	-0.02326	*
**Neighbourhood deprivation**																
Least deprived	Reference				Reference				Reference				Reference			
2nd least deprived	-0.012	-0.169	0.146		-0.083	-0.238	0.071		-0.16103	-0.39295	0.070885		-0.05841	-0.28716	0.170332	
3rd least deprived	-0.008	-0.176	0.159		-0.076	-0.246	0.094		-0.00067	-0.24291	0.241565		0.081456	-0.16224	0.325148	
2nd most deprived	0.132	-0.038	0.301		-0.038	-0.210	0.134		0.127894	-0.117	0.372784		0.15505	-0.08947	0.399565	
Most deprived	0.029	-0.135	0.193		-0.079	-0.245	0.087		-0.00799	-0.23651	0.220527		-0.05491	-0.28564	0.175828	
**Contextual factors**																
Housing inadequacy					0.009	-0.043	0.061						0.020844	-0.04672	0.088412	
Poor parental MH					0.033	0.024	0.041	***					0.031134	0.019234	0.043034	***
Lack of family support					0.031	0.006	0.057	*					0.001567	-0.03515	0.038284	
Parental concerns					0.034	0.001	0.067	*					0.015335	-0.02917	0.059845	
Challenges with education					0.067	0.042	0.092	***					0.083203	0.047708	0.118697	***
Perceived inadequacy of child's MH care					0.007	-0.022	0.036						0.008577	-0.03152	0.048673	
Limited outdoor time					0.110	0.051	0.170	***					0.111941	0.030394	0.193489	**
Difficulty with 'social distancing'					0.112	0.047	0.177	**					0.05543	-0.03682	0.147681	
**Comorbidities**																
ADHD																
ASD													0.249467	0.088134	0.4108	**
EmD													-0.25385	-0.53126	0.023554	
ID													-0.1129	-0.53597	0.310179	

	EMOTIONAL CHANGE															
	ASD								EmD							
	Demographic adjustment				Demographic and contextual adjustment				Demographic adjustment				Demographic and contextual adjustment			
	Coeff	95%CI		p	Coeff	95%CI		p	Coeff	95%CI		p	Coeff	95%CI		p
**Age at lockdown**	-0.02203	-0.05273	0.00866		-0.00959	-0.04216	0.022968		-0.00595	-0.0598	0.047904		-0.038374	-0.09897	0.022224	
**Sex**	-0.01254	-0.20952	0.184448		0.058964	-0.12919	0.247119		-0.05952	-0.34863	0.229593		-0.032747	-0.36467	0.299173	
**Ethnicity**																
White	Reference				Reference				Reference				Reference			
Black	0.202698	-0.07786	0.483258		0.222456	-0.0517	0.496613		-0.37992	-0.81416	0.054319		-0.375515	-0.86867	0.117641	
Asian	-0.1521	-0.98433	0.680137		-0.12601	-0.88108	0.629062		0.297593	-0.52005	1.115235		0.084906	-0.73505	0.904859	
Mixed	-0.09413	-0.40959	0.221322		0.025255	-0.28191	0.332418		-0.16932	-0.66203	0.3234		-0.040162	-0.58018	0.499854	
Other	-0.31453	-1.23177	0.602721		0.06382	-1.22457	1.352211		-0.16684	-1.32807	0.994381		-0.225477	-1.58206	1.131101	
Non-stated	-0.06023	-0.32329	0.202822		-0.10262	-0.35377	0.148525		-0.44582	-0.88463	-0.00702	*	-0.557114	-1.028	-0.08622	*
**Neighbourhood deprivation**																
Least deprived	Reference				Reference				Reference				Reference			
2nd least deprived	0.07321	-0.20721	0.353632		0.085605	-0.17981	0.351018		0.055072	-0.33926	0.449404		-0.10678	-0.52715	0.313593	
3rd least deprived	0.220118	-0.07989	0.520126		0.157715	-0.13781	0.45324		0.148347	-0.28987	0.586561		-0.046335	-0.53397	0.441301	
2nd most deprived	0.183428	-0.12107	0.487927		0.092599	-0.2018	0.387		0.393268	-0.02972	0.816259		0.097042	-0.36941	0.563494	
Most deprived	0.101205	-0.16975	0.372162		0.003211	-0.26097	0.267391		0.296686	-0.18166	0.775031		0.161085	-0.35922	0.681388	
**Contextual factors**																
Housing inadequacy					-0.02443	-0.11019	0.061325						0.034705	-0.12212	0.191531	
Poor parental MH					0.037049	0.022651	0.051447	***					0.03825	0.014432	0.062068	**
Lack of family support					0.026987	-0.01704	0.071014						0.009672	-0.0673	0.086646	
Parental concerns					0.057249	0.00283	0.111669	*					0.039117	-0.05265	0.130887	
Challenges with education					0.053737	0.011869	0.095605	*					-0.003594	-0.06899	0.061804	
Perceived inadequacy of child's MH care					-0.00057	-0.04743	0.046285						0.048835	-0.04705	0.144715	
Limited outdoor time					0.110199	0.005174	0.215223	*					0.243515	0.062816	0.424214	**
Difficulty with 'social distancing'					0.130387	0.024894	0.23588	*					0.241145	0.028524	0.453766	*
**Comorbidities**																
ADHD					0.148221	-0.03838	0.334823						0.050169	-0.3388	0.439138	
ASD													0.032816	-0.38098	0.446609	
EmD					-0.1711	-0.48735	0.145139									
ID					-0.21913	-0.54288	0.104616						-0.207458	-1.54487	1.12995	

Number of observations:

Whole sample: emotional change, demographic adjustment N=1502, demographic and contextual adjustment N=1290

ADHD: emotional change, demographic adjustment N=640, demographic and contextual adjustment N=540

ASD: emotional change, demographic adjustment N=471, demographic and contextual adjustment N=406

EmD: emotional change, demographic adjustment N=266 demographic and contextual adjustment N=229

Table S7. Adjusted regressions for total behavioral score. This table shows the results of the linear regression analyses progressively adjusting for pre-Covid socio-demographic characteristics and Covid-related contextual factors. In the fully adjusted models for the three main diagnostic groups, we also included co-occurrent disorders, if present, as covariates. Numbers of observations are reported in footnotes.

TOTAL BEHAVIOURAL SCORE

	WHOLE SAMPLE								ADHD							
	Demographic adjustment				Demographic and contextual adjustment				Demographic adjustment				Demographic and contextual adjustment			
	Coeff	95%CI		p	Coeff	95%CI		p	Coeff	95%CI		p	Coeff	95%CI		p
**Age at lockdown**	-0.256	-0.319	-0.192	***	-0.248	-0.307	-0.190	***	-0.222	-0.317	-0.127	***	-0.178	-0.272	-0.083	***
**Sex**	-0.840	-1.249	-0.431	***	-0.359	-0.722	0.005		-0.202	-0.869	0.466		-0.027	-0.642	0.589	
**Ethnicity**																
White	Reference				Reference				Reference				Reference			
Black	-0.891	-1.456	-0.327	**	-0.738	-1.253	-0.223	**	-0.570	-1.414	0.275		-0.623	-1.416	0.171	
Asian	-0.005	-1.691	1.682		-0.684	-2.068	0.701		-0.640	-4.414	3.133		-0.346	-3.586	2.895	
Mixed	-0.561	-1.226	0.105		-0.533	-1.119	0.053		0.339	-0.675	1.353		0.141	-0.803	1.086	
Other	-1.153	-2.841	0.535		0.062	-1.474	1.599		-1.476	-3.766	0.813		0.133	-1.882	2.148	
Non-stated	-0.412	-0.972	0.148		-0.503	-1.001	-0.005	*	-0.551	-1.343	0.242		-0.747	-1.482	-0.013	*
**Neighbourhood deprivation**																
Least deprived	Reference				Reference				Reference				Reference			
2nd least deprived	-0.119	-0.704	0.465		-0.286	-0.794	0.222		-1.113	-2.022	-0.204	*	-0.907	-1.737	-0.076	*
3rd least deprived	0.211	-0.413	0.834		0.116	-0.444	0.676		0.035	-0.914	0.984		0.171	-0.714	1.055	
2nd most deprived	0.852	0.221	1.483	**	0.323	-0.242	0.888		0.051	-0.909	1.010		-0.204	-1.091	0.684	
Most deprived	0.075	-0.535	0.686		-0.287	-0.834	0.260		-0.449	-1.345	0.447		-0.818	-1.656	0.019	
**Contextual factors**																
Housing inadequacy					0.161	-0.010	0.333						0.084	-0.161	0.329	
Poor parental MH					0.154	0.126	0.182	***					0.174	0.131	0.217	***
Lack of family support					0.175	0.090	0.260	***					0.038	-0.095	0.171	
Parental concerns					-0.006	-0.114	0.102						0.054	-0.107	0.215	
Challenges with education					0.513	0.431	0.594	***					0.407	0.278	0.535	***
Perceived inadequacy of child's MH care					0.177	0.082	0.272	***					0.173	0.027	0.318	*
Limited outdoor time					-0.049	-0.245	0.147						-0.040	-0.336	0.255	
Difficulty with ‘social distancing’					0.447	0.234	0.660	***					0.559	0.225	0.894	**
**Comorbidities**																
ADHD																
ASD													0.673	0.087	1.258	*
EmD													-0.514	-1.520	0.491	
ID													-0.050	-1.583	1.484	

	ASD								EmD							
	Demographic adjustment				Demographic and contextual adjustment				Demographic adjustment				Demographic and contextual adjustment			
	Coeff	95%CI		p	Coeff	95%CI		p	Coeff	95%CI		p	Coeff	95%CI		p
**Age at lockdown**	-0.316	-0.430	-0.203	***	-0.231	-0.347	-0.115	***	-0.263	-0.431	-0.094	**	-0.334	-0.499	-0.169	***
**Sex**	-0.088	-0.815	0.639		0.064	-0.607	0.735		0.093	-0.810	0.995		0.657	-0.246	1.560	
**Ethnicity**																
White	Reference				Reference				Reference				Reference			
Black	-0.767	-1.801	0.268		-0.589	-1.565	0.388		-0.886	-2.241	0.469		-0.906	-2.248	0.436	
Asian	0.384	-2.684	3.453		0.629	-2.060	3.319		1.163	-1.389	3.715		0.413	-1.818	2.644	
Mixed	-1.379	-2.542	-0.215	*	-1.188	-2.282	-0.094	*	-1.426	-2.963	0.112		-1.172	-2.641	0.297	
Other	-1.163	-4.546	2.219		1.239	-3.349	5.828		1.021	-2.604	4.645		0.158	-3.533	3.849	
Non-stated	-0.664	-1.634	0.307		-0.695	-1.590	0.199		-0.771	-2.140	0.599		-0.720	-2.002	0.561	
**Neighbourhood deprivation**																
Least deprived	Reference				Reference				Reference				Reference			
2nd least deprived	0.117	-0.919	1.152		0.014	-0.932	0.961		0.508	-0.723	1.739		-0.063	-1.206	1.081	
3rd least deprived	1.372	0.264	2.479	*	0.524	-0.529	1.578		0.723	-0.645	2.091		0.627	-0.700	1.954	
2nd most deprived	0.536	-0.588	1.660		0.206	-0.844	1.255		1.756	0.436	3.077	**	0.816	-0.453	2.085	
Most deprived	-0.282	-1.283	0.718		-0.868	-1.811	0.075		0.886	-0.607	2.379		0.297	-1.119	1.713	
**Contextual factors**																
Housing inadequacy					0.060	-0.245	0.366						0.221	-0.206	0.647	
Poor parental MH					0.149	0.098	0.201	***					0.160	0.096	0.225	***
Lack of family support					0.122	-0.035	0.279						-0.019	-0.229	0.190	
Parental concerns					0.117	-0.077	0.311						0.008	-0.241	0.258	
Challenges with education					0.347	0.197	0.496	***					0.481	0.303	0.658	***
Perceived inadequacy of child's MH care					0.128	-0.038	0.295						0.212	-0.049	0.473	
Limited outdoor time					0.170	-0.204	0.544						0.302	-0.189	0.794	
Difficulty with ‘social distancing’					0.447	0.072	0.823	*					0.539	-0.040	1.117	
**Comorbidities**																
ADHD					0.888	0.223	1.553	**					0.736	-0.322	1.795	
ASD													0.424	-0.701	1.550	
EmD					-0.787	-1.913	0.340									
ID					-0.101	-1.254	1.052						-0.824	-4.463	2.815	

Number of observations:

Whole sample: total behavioral score, demographic adjustment N=1502, demographic and contextual adjustment N=1290

ADHD: total behavioral score, demographic adjustment N=639, demographic and contextual adjustment N=539

ASD: total behavioral score, demographic adjustment N=470, demographic and contextual adjustment N=405

EmD: total behavioral score, demographic adjustment N=266 demographic and contextual adjustment N=229

Table S8. Adjusted regressions for behavioral change. This table shows the results of the linear regression analyses progressively adjusting for pre-Covid socio-demographic characteristics and Covid-related contextual factors. In the fully adjusted models for the three main diagnostic groups, we also included co-occurrent disorders, if present, as covariates. Numbers of observations are reported in footnotes.

	BEHAVIOURAL CHANGE																															
	WHOLE SAMPLE								ADHD								ASD								EmD							
	Demographic adjustment				Demographic and contextual adjustment				Demographic adjustment				Demographic and contextual adjustment				Demographic adjustment				Demographic and contextual adjustment				Demographic adjustment				Demographic and contextual adjustment			
	Coeff	95%CI		p	Coeff	95%CI		p	Coeff	95%CI		p	Coeff	95%CI		p	Coeff	95%CI		p	Coeff	95%CI		p	Coeff	95%CI		p	Coeff	95%CI		p
**Age at lockdown**	-0.030	-0.046	-0.013	***	-0.029	-0.046	-0.012	***	-0.023	-0.047	0.001		-0.020	-0.046	0.006		-0.050	-0.081	-0.019	**	-0.026	-0.058	0.006		-0.007	-0.056	0.041		-0.028	-0.081	0.026	
**Sex**	-0.158	-0.265	-0.052	**	-0.065	-0.169	0.040		-0.043	-0.212	0.126		-0.035	-0.202	0.133		0.026	-0.172	0.224		0.028	-0.158	0.215		-0.108	-0.371	0.155		-0.136	-0.429	0.158	
**Ethnicity**																																
White	Reference				Reference				Reference				Reference				Reference				Reference				Reference				Reference			
Black	-0.044	-0.191	0.103		-0.032	-0.180	0.117		-0.146	-0.359	0.068		-0.229	-0.445	-0.013	*	-0.026	-0.308	0.257		-0.017	-0.288	0.255		-0.028	-0.424	0.368		0.109	-0.326	0.545	
Asian	-0.237	-0.677	0.202		-0.398	-0.796	0.001	*	-0.261	-1.217	0.696		-0.266	-1.148	0.616		-0.349	-1.185	0.488		-0.260	-1.009	0.488		0.079	-0.666	0.824		-0.068	-0.794	0.657	
Mixed	-0.073	-0.246	0.100		-0.086	-0.255	0.083		0.038	-0.219	0.295		-0.082	-0.339	0.175		-0.104	-0.421	0.213		0.013	-0.291	0.318		0.113	-0.336	0.562		0.292	-0.185	0.770	
Other	-0.342	-0.782	0.097		-0.275	-0.717	0.168		-0.403	-0.983	0.177		-0.174	-0.722	0.375		0.501	-0.421	1.423		0.652	-0.625	1.929		0.434	-0.624	1.493		0.103	-1.097	1.302	
Non-stated	-0.084	-0.229	0.062		-0.109	-0.253	0.034		-0.126	-0.327	0.074		-0.160	-0.359	0.040		-0.072	-0.337	0.192		-0.070	-0.319	0.178		-0.413	-0.813	-0.013	*	-0.295	-0.711	0.121	
**Neighbourhood deprivation**																																
Least deprived	Reference				Reference				Reference				Reference				Reference				Reference				Reference				Reference			
2nd least deprived	-0.001	-0.153	0.151		-0.066	-0.212	0.080		-0.221	-0.451	0.009		-0.158	-0.383	0.067		0.137	-0.145	0.419		0.073	-0.190	0.336		0.092	-0.266	0.450		-0.040	-0.410	0.330	
3rd least deprived	-0.022	-0.184	0.140		-0.054	-0.215	0.107		-0.025	-0.265	0.215		0.007	-0.233	0.247		0.216	-0.085	0.518		0.057	-0.235	0.350		0.029	-0.369	0.427		0.012	-0.418	0.442	
2nd most deprived	0.121	-0.043	0.285		-0.033	-0.195	0.130		0.054	-0.189	0.297		-0.027	-0.268	0.214		0.215	-0.091	0.521		0.147	-0.145	0.438		0.279	-0.105	0.664		0.105	-0.306	0.516	
Most deprived	0.022	-0.137	0.180		-0.071	-0.229	0.086		-0.017	-0.242	0.209		-0.052	-0.279	0.175		0.094	-0.178	0.367		0.005	-0.256	0.267		0.142	-0.293	0.578		-0.003	-0.462	0.457	
**Contextual factors**																																
Housing inadequacy					0.025	-0.024	0.074						0.066	-0.001	0.132						0.021	-0.064	0.106						-0.003	-0.142	0.135	
Poor parental MH					0.033	0.025	0.041	***					0.029	0.017	0.041	***					0.028	0.013	0.042	***					0.042	0.021	0.063	***
Lack of family support					0.039	0.014	0.063	**					0.017	-0.019	0.054						0.057	0.013	0.100	*					0.017	-0.051	0.085	
Parental concerns					0.022	-0.009	0.053						0.029	-0.015	0.073						0.030	-0.024	0.084						0.011	-0.070	0.092	
Challenges with education					0.082	0.058	0.105	***					0.076	0.041	0.111	***					0.068	0.026	0.109	**					0.042	-0.015	0.100	
Perceived inadequacy of child's MH care					-0.005	-0.032	0.022						0.011	-0.029	0.050						-0.017	-0.063	0.030						-0.041	-0.125	0.044	
Limited outdoor time					0.034	-0.022	0.090						0.037	-0.044	0.117						0.105	0.001	0.209	*					0.133	-0.027	0.292	
Difficulty with ‘social distancing’					0.086	0.025	0.147	**					0.040	-0.051	0.131						0.090	-0.015	0.195						0.178	-0.009	0.365	
**Comorbidities**																																
ADHD																					0.173	-0.012	0.358						0.019	-0.322	0.361	
ASD													0.165	0.006	0.325	*													-0.050	-0.415	0.315	
EmD													-0.241	-0.512	0.029						-0.218	-0.532	0.095									
ID													-0.294	-0.711	0.124						-0.209	-0.530	0.111						-0.334	-1.517	0.850	

Number of observations:

Whole sample: behavioral change, demographic adjustment N=1505, demographic and contextual adjustment N=1291

ADHD: behavioral change, demographic adjustment N=643, demographic and contextual adjustment N=541

ASD: behavioral change, demographic adjustment N=471, demographic and contextual adjustment N=406

EmD: behavioral change, demographic adjustment N=267 demographic and contextual adjustment N=230

Table S9. Table S5. Sensitivity analysis. When we contrasted the three main diagnostic groups as predictor of each mental health outcome in unadjusted and fully adjusted regression models, most of the observed differences between diagnostic groups held independently from contextual and socio-demographic factors.

	**Total emotional difficulties**	**Emotional change**	**Total behavioural difficulties**	**Behavioural change**
**Unadjusted models**				
ADHD - ASD	F(1,1071)=19.43, p<0.001	F(1,1074)=8.47, p=0.003	ns	ns
ADHD - EmD	F(1,1071)=10.11, p=0.001	F(1,1074)=6.78, p=0.009	F(1,1073)=30.55, p<0.001	F(1,1077)=19.21, p<0.001
ASD -EmD	ns	F(1,1074)=6.78, p=0.009	F(1,1073)=42.39, p<0.001	F(1,1077)=28.18, p<0.001
**Fully adjusted models**				
ADHD - ASD	F(1,877)=7.54, p=0.006	F(1,879)=5.73, p=0.016	ns	ns
ADHD - EmD	ns	ns	F(1,878)=10.40, p=0.001	ns
ASD -EmD	ns	F(1,879)=12.76, p<0.001	F(1,878)=17.45, p<0.001	F(1,880)=7.29, p=0.007

Table S10. Education and mental health. This table shows the results of the linear regressions testing the associations between education engagement/enjoyment and emotional/behavioral change. Poor education engagement/enjoyment was associated with worse emotional and behavioral change as compared to before the pandemic in the whole sample and in the three main diagnostic groups (apart from emotional change for those with EmD). Please note that for education engagement/enjoyment, higher scores reflect lower engagement/enjoyment whilst for emotional/behavioral change higher scores reflect greater change, thus positive coefficients indicate negative associations.

	**WHOLE SAMPLE**	**ADHD**	**ASD**	**EmD**
	coeff (p-value)	coeff (p-value)	coeff (p-value)	coeff (p-value)
**EDUCATION ENGAGEMENT**				
Emotional change	0.16 (p<0.001)	0.15 (p<0.001)	0.15 (p<0.001)	0.05 (p=0.39)
Behavioural change	0.19 (p<0.001)	0.16 (p<0.001)	0.17 (p<0.001)	0.11 (p=0.04)
**EDUCATION ENJOYMENT**				
Emotional change	0.23 (p<0.001)	0.19 (p<0.001)	0.20 (p<0.001)	0.17 (p=0.02)
Behavioural change	0.23 (p<0.001)	0.17 (p<0.001)	0.23 (p<0.001)	0.18 (p=0.005)

4. LIST OF ABBREVIATIONS

ADHD= Attention Deficit Hyperactivity Disorder; API= Application Programming Interface; ASD= Autism Spectrum Disorder; CAMHS= Child and Adolescent Mental Health Services; CRIS= Clinical Record Interactive Search; COPI= Control of Patient Information Regulations; CYP= children and young people; CYPHER= Maudsley Child and Young People Health & Experience Research; EHR= electronic health record; EmD= emotional disorders; ePJS= electronic Patient Journey System; ICD-10= International Classification of Diseases; project ID=identity; ID= intellectual disability; IMD= Index of Multi-deprivation; MH= mental health; ns= not significant; OCD= Obsessive-Compulsive Disorder; ONS= Office of National Statistics; PII= personal identifiable information; PTSD= Post-Traumatic Stress Disorder; SLaM=South London and Maudsley NHS Foundation Trust; URL=Uniform Resource Locators; YP=young people.

5. REFERENCES

1. Samji, H., et al., *Review: Mental health impacts of the COVID-19 pandemic on children and youth - a systematic review.* Child and Adolescent Mental Health., 2021.
2. Downs, J., et al., *Negative Symptoms in Early-Onset Psychosis and Their Association With Antipsychotic Treatment Failure.* Schizophr Bull, 2018.
